# A Hyperbolic Sum Rule for Probability: Solving Recursive (“Chicken and Egg”) Problems

**DOI:** 10.3390/e27040352

**Published:** 2025-03-28

**Authors:** Michael C. Parker, Chris Jeynes, Stuart D. Walker

**Affiliations:** 1 School of Computer Sciences & Electronic Engineering, University of Essex, Colchester CO4 3SQ, UK; stuwal@essex.ac.uk; 2 Independent Researcher, Tredegar NP22 4LP, UK

**Keywords:** QGT, entropy, Venn diagram, Bayes’ Theorem, AI, ML, DSP

## Abstract

We prove that the probability of “*A* or *B*”, denoted as *p*(*A* or *B*), where *A* and *B* are events or hypotheses that may be recursively dependent, is given by a “Hyperbolic Sum Rule” (**HSR**), which is relationally isomorphic to the hyperbolic tangent double-angle formula. We also prove that this HSR is Maximum Entropy (**MaxEnt**). Since this recursive dependency is commutative, it maintains the symmetry between the two events, while the recursiveness also represents temporal symmetry within the logical structure of the HSR. The possibility of recursive probabilities is excluded by the “Conventional Sum Rule” (**CSR**), which we have also proved to be MaxEnt (with lower entropy than the HSR due to its narrower domain of applicability). The concatenation property of the HSR is exploited to enable analytical, consistent, and scalable calculations for multiple hypotheses. Although they are intrinsic to current artificial intelligence and machine learning applications, such calculations are not conveniently available for the CSR, moreover they are presently considered intractable for analytical study and methodological validation. Where, for two hypotheses, we have *p*(*A*|*B*) > 0 and *p*(*B*|*A*) > 0 together (where “*A*|*B*” means “*A* given *B*”), we show that *either* {*A*,*B*} is independent *or* {*A*,*B*} is recursively dependent. In general, recursive relations cannot be ruled out: the HSR should be used by default. Because the HSR is isomorphic to other physical quantities, including those of certain components that are important for digital signal processing, we also show that it is as reasonable to state that “*probability is physical*” as it is to state that “*information is physical*” (which is now recognised as a truism of communications network engineering); probability is *not* merely a mathematical construct. We relate this treatment to the physics of Quantitative Geometrical Thermodynamics, which is defined in complex hyperbolic (Minkowski) spacetime.

## 1. Introduction I: Probabilities

### 1.1. Overview

Surely how to sum probabilities has been known for generations? The formula for “*p*(*A* or *B*)” (“the probability of either or both instances of {*A*,*B*} happening”, where “or” is the logico-probabilistic operator: see Equation (2b), below), has been well known since John Venn published his textbook in 1881; the formula was known much earlier (as mentioned by Leonhard Euler in 1768, for instance). Why should we want to replace or improve upon the canonical “Conventional Sum Rule” (**CSR**) expression?

The problem is that the CSR is not valid where the probabilities of the events of *A* and *B* are *recursively* dependent on each other, or where their causal priority (temporal or logical) is indeterminate. However, the artificial intelligence (AI) and machine learning (ML) applications widely employed today regularly address complex problems involving multiple hypotheses with multiple conditionalities, including recursive ones. Thus, more general methods are needed.

It turns out that the CSR is a special case; using Bayesian methods to derive the general case results in a “Hyperbolic Sum Rule” (**HSR**). We start from the seminal work of Richard Cox (1946 [1]) and Edwin Jaynes (2003 [2], 1982 [3]), presenting the HSR Theorem in Section 3.1 (Equation (12)), and further summarise the technical relations in Section 3.3 (as proved in the Appendix A).

Bayesian methods make explicit the physicality of *probability*, in particular, the fact that *agents* are always involved where unbiassed estimates of the probabilities of events or hypotheses are required. Then, the value of “Maximum Entropy” (**MaxEnt**) techniques becomes evident. We prove that the HSR is MaxEnt (in Appendix A.3) using standard Lagrange multiplier methods (see, for example, Caticha 2008 [4]).

What sense does it make to say that a *probability sum rule* is “MaxEnt”? What rôle could *thermodynamics* be thought to play in calculating probabilities (for entropy is a thermodynamic quantity)? It is usually thought that thermodynamics is primarily a way of handling statistical ensembles, but that this is not the case can be seen from the success of “Quantitative Geometrical Thermodynamics” (**QGT**) in calculating *geometrical* parameters of nuclear, molecular and cosmic structures (DNA and the Milky Way: Parker & Jeynes 2019 [5]; nuclear dimensions of helium isotopes: Parker et al., 2022 [6]). Note also that QGT is defined in a *hyperbolic* space [5], underlining the explicit link with a *hyperbolic* sum rule for probabilities.

The point is that the probability of a given event can always be thought to be drawn from a probability distribution, and to obtain an unbiassed estimate of this probability one uses a Maximum Entropy distribution. But to specify a distribution, one gives its functional form so that one can ascribe an entropy to the function. To say that the HSR is MaxEnt is to say that the resulting probabilities are the best (the most unbiassed) estimates available, given the current knowledge. It is important to point out that the mathematical objects resulting from the HSR described here are still regular *probabilities*, with real values between 0 and 1 (inclusive), provided that the value inputs into the formulae are also regular probabilities. This proviso is, of course, the same for both the CSR and the product rule (Bayes’ Theorem).

The motivation for the HSR is that the artificial intelligence (**AI**) and machine learning (**ML**) applications widely employed today regularly use Bayesian methods to address complex problems involving multiple hypotheses with multiple conditionalities. We elaborate on this point briefly in Section 4.4. Of course, these problems do not exclude recursive relations. But the CSR is not valid in the presence of recursion, and therefore, such problems need more general analytical methods. Moreover, separately, digital signal processing (**DSP**) methods are formally closely related to these new methods in probability (see the further discussion in Section 4.3).

The paper is extensive because it introduces a generalised (and far-reaching) sum rule for probabilities, and is structured as follows: In Section 1, we outline the very extensive prior work relating to how we think about *probabilities*, and in Section 2, we do the same for the Conventional Sum Rule. We discuss the limitations of the Venn diagram in Section 2.2 and *informally* derive the HSR in Section 2.3 (Equation (8)) before *formally* deriving it in Section 3.1 (Equation (12)). This informal approach is presented because a physical appreciation of this far-reaching result is needed to promote the DSP and AI applications we have in mind, as indicated in Section 4. Finally, we summarise (Section 5) and conclude (Section 6).

### 1.2. Probability in Science

Probability has always been extraordinarily difficult to tie down. Edwin Jaynes made fundamental contributions to the subject, and in his monograph (“*Probability Theory: The Logic of Science*” [2]) spends two chapters on its principles and elementary applications, commenting on its ‘weird’ and ‘complicated’ history. Indeed, Jaynes’ motivation was aimed at helping the interested reader who already has “*a previous acquaintance with probability and statistics*” to essentially “*unlearn*” much of what they may have previously learned!

The ubiquity (and longevity) of fallacies in the use of statistics indicates its difficulty (on the misuse of the “*p*-value” see, for example, Goodman 2008 [7] and Halsey 2019 [8]; and on the persistence of fallacies see Smaldino and McElreath 2016 [9]). A significant part of the problem may be related to the fact that the “probability” of an event is apparently not a property solely of “external reality”: since there must be *someone* assessing the probability, it must be a function of not only *what* information already exists about the event but also *who* knows it. The fact that our estimates of the probability of some event invariably involve our prior knowledge, combined with the fact that all knowledge ultimately involves the properties of recursive statements (by Gödel’s Theorem [10]; this point has recently been elaborated by Jeynes et al. [11], and Jeynes and Parker [12]) mean that some part of this difficulty must be due to the neglecting, in current (simplified) treatments, of recursive dependencies (such as the “Chicken and Egg” problem, on which see Section 2.3 below).

In 1946, R.T.Cox observed, acutely, that the concept of *probability* mixes two separate concepts (“*the idea of frequency in an ensemble and the idea of reasonable expectation*” [1]), which are now represented by two schools usually called the “frequentists” and the “Bayesians”. We point out that Enßlin et al. [13] comment that, “*The different views on probabilities of frequentists and Bayesians are addressed by … Allen Caldwell [14] [who] builds a bridge between these antagonistic camps by explaining frequentists’ constructions in a Bayesian language*”, and we will assume that Caldwell is right and therefore that we can ignore the philosophical distinction between the frequentists and the Bayesians.

Building on Cox’s fundamental work we will here derive a rigorous treatment of recursive probability which is of general applicability. In particular, we will treat probability as a *physical quantity* grounded in hyperbolic Minkowski spacetime, and obeying all the appropriate physical laws. The close relation of *probability* to the new Quantitative Geometrical Thermodynamics [5] (**QGT**, which constructs the quantity *info-entropy* by treating the bases of *information* and *entropy* as Hodge duals) is due to the fact that the (hyperbolic) *entropic velocity q*′ ≡ d*q*/d*x* in QGT is dimensionless and has properties akin to a probability (0 ≤ *q*′ ≤ 1); noting that *q*′ is isomorphic to the more familiar kinematic velocity $\overset{\;.}{x}$ ≡ d*x*/d*t* (0 ≤ $\overset{\;.}{x}$ ≤ *c*, where *c* is the speed of light) in hyperbolic (Minkowski) spacetime (see [5]), and which also obeys the well-known velocity addition theorem of Special Relativity (see Equation (1b)).

The concept of probability as a *physical* quantity (and obeying *physical* laws) is as fundamental as the well-known statement by Rolf Landauer that “*Information is Physical*” [15].

We are used to treating both *probability* and *information* anthropomorphically (that is, depending on what you or I might be expected to know): here we will establish an impersonal sense of *probability*, in the same way that Landauer insisted on the (impersonal) *Shannon entropy* sense of his *Information*. Note that each of information and entropy both use and require probabilistic quantities in their fundamental definitions; and also note that the consequences of treating *information* as being a quantity as physical as is *energy* (for example) has led to important insights, and not only into basic engineering problems of the global internet (for an example of which see Parker et al., 2015 [16]). We expect similar advances to follow from recognising *probability* as also being an equally physical quantity.

The relationship between probability and the quantification of information using the Shannon entropy is well understood mathematically, but its interpretation as a physical theory has been convoluted. Although the Shannon entropy was quickly recognised as important, Edwin Jaynes’ formulation of Maximum Entropy (**MaxEnt**) theory, where the Shannon metric plays a key role, was initially controversial and took some decades to achieve acceptance (see Jaynes’ 1978 summary [17]). However, MaxEnt as a powerful scientific and engineering tool has helped considerably to underpin the physicality of *information*, and therefore also acts as a support to the underlying assertion of this paper (paraphrasing Landauer) that “*Probability is Physical*”. We will also explore the implications of this assertion.

Since the concept of MaxEnt will be centrally important in this work, it is worth adding Jaynes’ authoritative definition (1982) [3]: “*The MaxEnt principle, stated most briefly, is: when we make inferences based on incomplete information, we should draw them from that probability distribution that has the maximum entropy permitted by the information we do have*”. Note that distributions are described by functions, and therefore functions can also be said to be “MaxEnt”. We emphasise this since QGT applies specifically also to small systems (see, for example, Parker et al., 2022, [6]) for which statistical mechanics methods cannot apply. It is worth mentioning that this ambiguity between “distributions” and “functions” is analogous to that between “frequentists” and “Bayesians”. The same thing may be described in various ways, and different things may have the same mathematical representation.

### 1.3. Probability Is Physical

Torsten Enßlin’s treatment [18] of information as a field is interesting in this context: he considers that a “physical field has an infinite number of degrees of freedom since it has a field value at each location of a continuous space” where he is specifically considering imaging problems of astrophysical datasets. Enßlin et al. [13] treat information informally as an anthropomorphic concept: to the question, “What is information?” they answer, “Anything that changes our minds and states of thinking!” But here, we will treat information (and probability) as physical quantities, not as anthropomorphic concepts; and especially noting that infinite quantities are inimical to physical phenomena. In particular, in our QGT treatment, information is formally defined as a physical quantity (albeit in terms making full use of the properties of analytical continuation, which is itself closely related to fundamental physical precepts such as causality [19] and square-integrability [20] ensuring finite physical quantities [21]), so that the number of degrees of freedom is finite and may be very small, as is observed for the geometrical entropy of isotopes of the helium nucleus [6]. Such results for information were already pointed out by Parker and Walker (2004) [22] who investigated the residues of a meromorphic function (that is, a function analytic nearly everywhere) due to the presence of isolated singularities in the complex plane (singularities which are entirely analogous to particles in their behaviour); and who show that the information (entropy) of such a function is simply given by the sum of the residues.

This is immediately applicable to the Schrödinger equation: it is interesting that we will conclude (Equation (8)) that the appropriate **Sum Rule** for recursive probabilities has a **hyperbolic form**, and we will draw out the relation of this to the (hyperbolic) *entropic velocities* in the QGT formalism [23], in which the *entropic* Uncertainty Principle and *entropic* isomorphs of the Schrödinger equation may be derived from the *entropic* Liouville Theorem; all based on the Boltzmann constant as the relevant quantum of entropy. Of course, QGT is constructed in a hyperbolic (complex) Minkowski 4-space (see §18.4 in Roger Penrose’s “*Road to Reality*” [24]; as another pertinent example, Maxwell’s electro-magnetic field is a hyperbolic version of the Cauchy-Riemann Equations: see Courant and Hilbert [25] vol.II ch.III §2 Equation (8) *passim*).

The ramifications of this are very wide. Parker and Jeynes [5] have already shown the relevance of QGT to the stability and structure of spiral galaxies (using the properties of black holes), and also that *entropy production* (Π ≡ dS/d*t*) is conserved even in relativistic Maximum Entropy systems [26]. These issues up to now have been treated as problems in quantum gravity, and Matt Visser [27] very helpfully reviews conservative entropic forces (in Euclidean space and non-relativistically, although he comments that representing general relativity entropically should be possible). Note also that Visser suggests that the negative entropies that appear in his treatment can be regarded as *information*, citing Brillouin’s idea of “negentropy” which Parker and Jeynes [5] have shown to be a subtle misapprehension of the relation between information and entropy (which are actually Hodge duals).

We believe that much progress may be made by using the coherent formalism of QGT (defined in hyperbolic space) which has been shown to apply to both quantum mechanical and gravitational systems (that is, at all scales from sub-atomic to cosmic: see [5,6]), and since quantum mechanics is built on probabilities, the demonstration here that the general recursive sum rule for probabilities is hyperbolic is a significant conceptual regularisation. This conclusion is reinforced by Knuth’s demonstration [28] that: “*The sum and product rules, which are familiar from, but not unique to, probability theory, arise from the fact that logical statements form a distributive (Boolean) lattice, which exhibits the requisite symmetries*”. Moreover, Jaeger [29] reviews a variety of treatments, some of which involve theories of generalised probability, aimed at deriving quantum mechanics from information theory.

The basic isomorphism for the hyperbolic sum rule for probabilities (that we will prove, see Equation (12)) is the (purely mathematical) double-angle identity for the hyperbolic tangent function:

tanh(*a* + *b*) ≡ {tanh*a* + tanh*b*}/{1 + tanh*a*·tanh*b*} (1a)

Another interesting and very simple isomorphism is the well-known relativistic sum rule for velocities {*u*, *v*}, given by Jackson in his well-known textbook (in the context of a discussion of aberration and the Fizeau experiment; §11.4, Eq.11.28, [30]):

*w* ≡ Sum(*u*, *v*) *w/c* = {*u/c* + *v/c*}/{1 + *uv*/*c*^2^} (1b)

where *c* is the speed of light. Jackson comments that if *u* = *c* then also *w* = *c*, which is an “*explicit statement of Einstein’s second postulate*” (*c* is a constant).

This latter (Equation (1b)) is clearly physical (since *c* is involved) where the former (Equation (1a)) is a mathematical identity. Note also that in optics the basic formula for the two-layer Fabry–Perot cavity (etalon) is well-known [31]:

*r*_3_ = {*r*_2_ + *r*_1_Φ}/{1 + *r*_2_*r*_1_Φ} Φ ≡ exp(−2i*k*Δ*z*) (1c)

where the overall scattering (reflectivity) coefficient *r*_3_ is due to a pair of sequential Fresnel reflections (*r*_1_, *r*_2_) separated by a distance Δ*z* for a light ray of propagation constant *k*; and where we note that light is the physical phenomenon *par excellence* exhibiting the physics of Special Relativity within the context of hyperbolic (Minkowski) spacetime. Corzine et al. [32] demonstrate that this formula is closely related to the hyperbolic addition rule (Equation (1a)) specifically by using a hyperbolic tangent substitution, which dramatically simplifies the use of the formula in real (multilayer) cases—see Section 2.2 for additional and related discussion.

This approach has recently been supported in an interesting way by Skilling and Knuth [33], who conclude: “*it must be acknowledged, quantum theory works. So does probability. And the two are entirely mutually consistent*.” Their argument shows logical reasons why *probability* should be regarded as *physical*.

Skilling and Knuth claim to not be interested (for these purposes) in the distinction between ontology and epistemology. They say ([33], §3.5):

The ontology–epistemology divide is, for quantitation at least, a distinction without a difference. A bit of information carries no flag to inform us whether it was assigned by a conscious agent or by a mechanical switch. Our job in science is to make sense of our observations, not to indulge in empty disputation between isomorphic views. Our goal here is derivation of a calculus fit for general purpose. Ontology and epistemology share the same symmetries, the same rules, and the same assignments. So, they share a common calculus.

which is suggestive of Karen Barad’s (2007 [34]) insistence that the distinction between ontology and epistemology is not a real one, and therefore, speaking strictly we should refer to “*onto-epistemology*” ([34], p. 43). Skilling and Knuth also say this ([33], §1).

But, if our object can perturb a partner object, then by symmetry the partner object can also perturb our object. We could assign either role to either.

Our calculus, whatever it is, must be capable of representing such interactions … This insight that *interactions are basic* is the source of “quantum-ness”.

Again, this recalls Barad’s thesis that “*the primary ontological unit is the phenomenon*” ([34], p. 333). However, when Skilling and Knuth ([33], §4) say, “*We start with an identifiable object*”, this is directly contradicted by Barad, who asserts that “objects” do not have “*an inherent ontological separability*” ([34], p. 340); that is, strictly speaking, identifiable objects do not actually exist per se (since everything is entangled with everything else). But Skilling and Knuth are not aiming at philosophical precision, only at a demonstrable computability; for these purposes such fine distinctions do not matter. They are right to avoid metaphysical considerations in scientific work: although when wider social implications are important it may be necessary to consider the metaphysics (see, for example, [11,12]).

However, it turns out that the inescapable human dimension appears to be especially pronounced in *probability*, in the sense that the very *idea* of a probability entails one’s personal state of knowledge or ignorance (and Michael Polanyi insisted long ago that all knowledge is necessarily personal [35]). Howson and Urbach [36] have carefully explained why, although Bayesian (and Maximum Entropy) methods are fully (and helpfully) rational: “*there seems to be no way of ‘objectively’ defining prior probabilities … this is really no weakness [since] it allows expert opinion due weight, and is a candid admission of the personal element which is there in all scientific work*”. Assessing probabilities necessarily entails assessing uncertainties, and this must always involve some value judgments: although we may do our best to speak rationally about such judgments, it cannot be excluded that different people will (rationally) come to different conclusions.

Note that although scientists have a duty to argue rationally, non-scientists also normally behave rationally. Rationality is a property of humans, and only a rather small subset of humans are scientists.

### 1.4. Maximum Entropy

It is necessary to make a few initial simple remarks about Maximum Entropy (**MaxEnt**) methods to clarify the discussion. Jaynes said in 1957 [37]: “*The guiding principle is that the probability distribution over microscopic states which has maximum entropy subject to whatever is known, provides the most unbiased representation of our knowledge of the state of the system. The maximum-entropy distribution is the broadest one compatible with the given information; it assigns positive weight to every possibility that is not ruled out by the initial data.*” In our derivation here of the Hyperbolic Sum Rule we emphasise that the proper application of MaxEnt methods precludes the surreptitious introduction of tacit assumptions (“knowledge”). That is, *all* the “priors” (prior knowledge that conditions the data) must be stated explicitly (and our ignorance implies that some priors must sometimes be estimated, which may involve personal value judgments).

A fully Bayesian analysis requires that all prior knowledge is explicitly stated, including the “knowledge” that there is no knowledge available, in which case an “unbiassed” estimate is required. This is usually stated in terms of the “Principle of Indifference” (**PI**), but unfortunately, there is a set of (“Bertrand”) paradoxes which appear to invalidate the PI in some circumstances. But Parker and Jeynes [38] have resolved these paradoxes using QGT methods by supplying the missing prior information (in the form of the scale invariance condition).

In the Appendix A, we explore the Maximum Entropy (MaxEnt) properties of the HSR, including proof that it really is MaxEnt. We first show how to impose the MaxEnt criterion (Appendix A.1) using the Partition Function description, and then show (Appendix A.2) how other simple sum rules may be “MaxEnt” but are inadmissible since they do not always result in formal probabilities. We prove explicitly that the HSR is MaxEnt (Appendix A.3) and also generalise it for multiple recursive hypotheses (Appendix A.4). We show that the Conventional Sum Rule (**CSR**) is also MaxEnt (Appendix A.5) within its own domain of applicability, and we also generalise the CSR for multiple hypotheses. Both are MaxEnt, that is, neither the HSR nor the CSR ‘smuggle’ in inadvertent or implicit assumptions within their specified contexts; yet, the HSR encompasses a wider domain of physical application that includes the possibility of recursion between phenomena, whereas the CSR a priori excludes recursion (although there may still be various mutual dependencies). The HSR must, therefore, be used where the properties of the recursion are unknown; that is to say, a set of phenomena are known to be correlated, but the mechanism (or ordering) of causation is not known. The entropy of the HSR is shown to be, in general, higher than that of the CSR (Appendix A.5.3). Therefore, in a Bayesian analysis, the HSR contains fewer implicit assumptions or constraints than the CSR–this being a consequence of the HSR also allowing the possibility of recursion, whereas the CSR a priori excludes recursion.

Finally, in Appendix A.6, we show the immediate relevance of this treatment to digital signal processing, in particular, the handling of so-called “infinite impulse response” and “finite impulse response” filters.

It is important here to point out that the fact that an entity is Maximum Entropy does *not* mean that the entity has no structure (even though MaxEnt necessarily implies “maximum ignorance”). The reality is more nuanced. For example, we have shown that, *at a certain scale*, the alpha particle is a (MaxEnt) *unitary entity* (than which exists no simpler) [6]. But of course, at a *different* scale we may see the alpha’s constituent four nucleons (protons and neutrons).

Also, being MaxEnt does not preclude change: for example, free neutrons decay (with a half-life calculated ab initio from QGT by Parker and Jeynes 2023 [39]). The most extreme known entity that is MaxEnt is the black hole, and black holes necessarily grow (proved by Parker & Jeynes in 2021 [26] and confirmed in 2023 [40]). Nor does the fact that some entities are unconditionally stable mean that the Second Law does not apply to them. On the contrary! The matter radius of the alpha particle is correctly calculated ab initio from QGT [6].

It might be thought that MaxEnt applies only to *distributions*, but this is a misconception based on the idea that thermodynamic explanations are always statistical (requiring large systems). But Parker and Jeynes (2019) [5] have shown explicitly that *small* systems can be analysed with QGT methods, functionalising the system entropy and defining the *entropic* Lagrangian (whose integration over space is called the “Exertion”, *isomorphic* to the kinematic Action). The appropriate Euler–Lagrange equations exist to minimise the Exertion, such that the *Principle of Least Exertion* then applies (isomorphic to the ubiquitous *Principle of Least Action*). The *entropic* Hamiltonian is obtained from the *entropic* Lagrangian via the appropriate Legendre transformation, and whose integration over space yields the system entropy, which in turn is maximised via the same Euler–Lagrange equations, yielding a MaxEnt *function*.

The physical principle of Maximum Entropy embodies the (physical) Second Law of Thermodynamics. Both the CSR and the HSR are MaxEnt in their respective domains, and therefore, each also embodies important aspects of the Second Law. Moreover, the *probabilities* calculated by either Rule (each with its own particular domain of applicability) refer to the probability either of the events themselves or of our reasonable expectations; these are both physical things since both we and they are physical.

## 2. Introduction II: Sum Rules

### 2.1. The Conventional Sum Rule (I)

Consider *p*(*A*) which is a representation of either “*the probability of event A occurring*” or, nearly equivalently (logically speaking), “*the probability of hypothesis A being true*”. Of course, hypotheses and events are entirely different things, but for convenience, we will here usually speak of “events” without any loss of generality (ignoring the philosophical frequentist/Bayesian distinction, see Section 1.2).

The probability that *A* or *B* will happen is conventionally given (by the “Conventional Sum Rule”, **CSR**) as the sum of the separate probabilities that *A* and *B*, respectively, will each happen, minus the probability that both of {*A*,*B*} will happen together, (in general, {*A*,*B*} are also not independent of each other):

*p*(*A* or *B*) = *p*(*A*) + *p*(*B*) − *p*(*A* and *B*) (2a)

This is simply extended to the conditional case treating the probability of *A* or *B* given some conditionality *C*:

*p*(*A* or *B*|*C*) = *p*(*A*|*C*) + *p*(*B*|*C*) − *p*(*AB*|*C*) (2b)

where *A* and *B* are two events, conditional either on each other or some other event(s); in other words, in this case *C* represents all the possible conditionalities, including the mutual conditionalities of {*A*,*B*}; that is, *p*(*A*|*B*), *p*(*B*|*A*) (“the probability of *A* given *B*”, and conversely). Equation (2b) represents the CSR. But if {*A*,*B*} are recursively dependent (the “chicken and egg” scenario) Equation (2b) is invalid since *C* either cannot be specified explicitly or cannot be specified at all.

Intrinsic to Equation (2a,b) is also the corresponding “Product Rule”, more commonly known as Bayes’ Theorem:

*p*(*A* and *B*|C) ≡ *p*(*AB*|*C*)             = *p*(*A*|*BC*) *p*(*B*|*C*)           = *p*(*B*|*AC*) *p*(*A*|*C*) (2c)

Here, Bayes’ Theorem appears in the latter two expressions of Equation (2c), which yield the same result since Bayes’ Theorem is commutative: *p*(*AB*) = *p*(*BA*). In particular, Bayes’ Theorem represents a *choice* as to which of the possible conditionalities should take priority. The logical AND function can be calculated in different ways according to which of the conditionalities is considered to be prior.

We emphasise: although we seek here an alternative form of the Sum Rule (Equation (2b)) that takes recursion into account, Bayes’ Theorem (Equation (2c)) remains valid.

Such recursive issues arise when we start considering which of the conditionalities is prior (an issue of causation): there appears to be a temporal dependence between the two conditionalities since one must surely occur earlier in time than the other. But note that Special Relativity teaches us that if two *spatially distinct* events are causally connected (that is, they lie within each other’s light cone) then temporal priority is always preserved whatever the frame of reference (FoR). However, if the events are not causally connected in spacetime, then for any FoR where the event of *A* occurs before that of *B*, another FoR exists where event *B* occurs before *A*. If causal connectedness cannot be determined, either due to the events being spacelike separated, or perhaps because no physical mechanism can be identified to causally connect the events, then there is an ambiguity as to which of the events is prior (and of course, correlation does not imply causation).

An example of a recursive system is the multilayer etalon represented by Equation (1c).

### 2.2. The Venn Diagram

Jaynes showed that the derivation of the product rule (Equation (2c)) from the basic laws of Boolean logic is straightforward [2]. However, performing the same derivation for the “Sum Rule” of probability is less obvious. In particular, it is worth noting that Jaynes specifically avoids the use of the Venn diagram as a means to justify Equation (2b), preferring to interpret propositions in terms not of sets but as probability distributions carrying incomplete information. It is useful to note in this context that there is intense current interest in how to handle incomplete (or erroneous) “information” in a formal Bayesian analysis (see, for example, Zhang et al. [41]).

**Figure 1:**
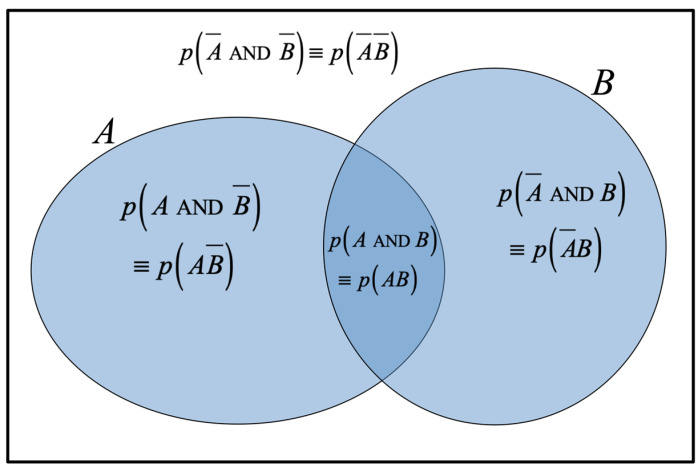
Classical Venn diagram showing the graphical relationship of logical quantities, with the overall enclosed (shaded) area representing the Conventional Sum Rule (CSR) for the probability of *A* OR *B*.

Jaynes therefore calls the Venn diagram a ‘useful device’ but comments that it can ‘*mislead*’ [our emphasis]. The Venn diagram is good for representing binary logical operations (see Figure 1) but, in general, correctly represents neither temporality (including aspects of causality or recursiveness) nor probability distributions. We add that it is also not obvious how the Venn diagram (or the CSR of Equation (2b)) could be usefully extended to include multiple hypotheses and/or conditionalities.

Jaynes’ derivation [2] of the “Product Rule” (Equation (2c)) does not need to involve de Morgan’s theorems (which follow from the rules of Boolean logic). But his derivation of the “Sum Rule” (Equation (2b)) adopts the original schema of Cox [1] to also define an auxiliary function *S* so that *v* ≡ *S*(*u*), where *u* ≡ *p*(*A*|*C*) such that *v* ≡ *p*($\bar{A}$|*C*) and shows that

*S*[*S*(*x*)] = *x* for 0 ≤ *x* ≤ 1 (3a)

that is, the function *S*(*x*) is involutory (self-reciprocal):

*S*(*x*) = *S*^−1^(*x*) (3b)

Using this reciprocal property of *S* to calculate inverse probabilities avoids the problem that there is no quotient operator in Boolean logic. In particular, for completeness we note that Cox employed the following particular function for *S* in his analysis:

*S*(*x*) = 1 − *x* (3c)

We can extend this treatment by generalising Cox’s function *S* to the function Σ:

(4a) $$\Sigma \left(x\right)=\frac{1-x}{1+x}$$

and here we point out some interesting properties of this new function: $\Sigma \left[\Sigma \left(x\right)\right]=x$; that is, Σ is involutive, $\Sigma \left(x\right)=\Sigma^{-1}\left(x\right)$. It is also doubly differentiable and the derivative is a continuous, monotonically decreasing function: $\partial \Sigma /\partial x=-2/{\left(1+x\right)}^{2}$ for real *x*, and 0 ≤ *x* ≤ 1. The inspiration for Equation (4a) comes from Cox’s original more general equation for *S* as another involutory function (see Equation (3) above) which is also doubly differentiable. In Cox’s case, he also considered the situations where *S* (and *x*) are raised by an integer power *m*, such that *S^m^*(*x*) = 1 − *x^m^* for integer *m*; which also conform to his mathematical requirements and Equation (3a,b). For even *m* > 0 this can be factored into the geometric series:

(1 − *x^m^*) ≡ (1 + *x*)(1 − *x* + *x*^2^ − *x*^3^ + … − *x^m^*^−1^) (4b)

with the series in parentheses summing to the quantity (1 + *x*)^−1^ as *m*→∞. Note that this treatment is quite general, such that *x* may be complex, although in this paper we only assume probabilities described by real *x*, and 0 ≤ *x* ≤ 1. However, for all *m* (with integer *m* ≥ 1), we can also write:

(1 − *x^m^*) ≡ (1 − *x*)(1 + *x* + *x*^2^ + *x*^3^ + … + *x^m^*^−1^) (4c)

Recognising that for even *m* the roots of *S^m^* lie symmetrically around the four quadrants of the unit circle centred on zero in the complex plane, allows us to additionally exploit the associated symmetry relation for the *n*th root, *x_n_* = −*x_m_*_/2+*n*_. It is important to emphasise that although results from complex analysis underpin this analysis, in the sum rules discussed in this paper we only ever assume (and employ) probability theoretical values for *x* where *x* is real and 0 ≤ *x* ≤ 1. Therefore, substituting *x_n_* = −*x_m_*_/2+*n*_ for the *m* − 1 terms of the series summation in Equation (4c), we can write:

(1 − *x^m^*) ≡ (1 − *x*)(1 − *x* + *x*^2^ − *x*^3^ + … − *x^m^*^−1^) (4d)

Applying this (counterintuitive) symmetry we see that Equation (4a) is simply the limit of Equation (4d) as *m*→∞. In this limit the distinction between even and odd *m* is insignificant. In any case, it is clear that although Cox’s first solution (using *m* = 1: the quantity *S* = 1 − *x*, see Equation (3c)) represents an important term in the factorisation of Σ, it is by no means the only available solution in the general case for the roots of the function Σ.

Note that although the intermediate Equation (4b,c,d) (and the implied complex analysis) are not used in the formal derivation of the HSR (Section 3.1 and Equation (12)), analogous complex quantities do make an appearance in the discussion of how the finite impulse response (FIR) filter offers a good exemplar of the CSR (sketched in Appendix A.6). The standard theory of such filters (which are important for digital signal processing applications) fundamentally relies on the theorems of complex analysis.

### 2.3. Recursion: Chicken and Egg

Dependencies can be logical or temporal. Causality (a temporal dependence) also implies the possibility of recursion: this is clear from the “Chicken and Egg” conundrum, on which see Simmons et al. [42] (who consider whether hummingbirds pollinate lots of species because there are lots of hummingbirds or whether there are lots of hummingbirds because there are lots of species to pollinate) or Harvey et al. [43] (who consider two forms of the recursive cyclic network to show how subtle recursion relations may be). The chicken entails a prior egg, which entails a prior chicken…

Formally: both *p*(*A*|*B*) > 0 and *p*(*B*|*A*) > 0 may be true together *either* if {*A*,*B*} are independent *or* if {*A*,*B*} are recursively dependent. However, recursion is *not* present if *C* in Equation (2b)—as in *p*(*A*|*C*)—is independent of either *A* or *B*; that is, if *p*(*C*|*A* or *B*) = *p*(*C*). Such expressions in Equation (2b) as *p*(*A*|*C*) imply that all dependencies of *A* are subsumed in *C*; that is, if *A* depends on *B* somehow, then there (usually) exists a *C* that will say this with little loss of generality (Hofer-Szabó et al. [44] show that Reichenbach’s “*common cause*” does not necessarily exist).

Here, we will informally derive the “Hyperbolic Sum Rule” that we will prove formally in Section 3.1.

There is an apparent dilemma (when applying Bayes’ Theorem) of having to make what appears to be an arbitrary decision as to which conditionality is the prior. Indeed, when considering the classic “Chicken and Egg” dilemma, having argued in one way, we undergo a *Gestalt* change and suddenly find ourselves equally arguing in the opposite manner! The recursiveness of this dilemma is self-evident; a different prior may be appropriate depending on where the analysis commences. Curiously, the case of the two-layer Fabry–Perot cavity (Equation (1c)) involves optical events that lie *on* each other’s light cone; that is to say, as a physical phenomenon, it is directly on the boundary of being causally connected or disconnected. In this case, an intrinsic ambiguity of priority exists. If either may be prior then neither must be.

That is, for a recursive dependency of {*A*,*B*}, *p*(*A*|*B*) = *p*(*A*) and by symmetry, we also must have *p*(*B*|*A*) = *p*(*B*). This is a surprising result since {*A*,*B*} being independent *also* entails *p*(*A*|*B*) = *p*(*A*): that is to say, the condition “*p*(*A*|*B*) = *p*(*A*)” may entail *either* “{*A*,*B*} are independent” *or* “{*A*,*B*} are recursively dependent”.

In any case, issues of the probability of events and their priority are frequently set within a spacetime context, with the associated physical implications. One of the aims of this paper is to establish a solution to this dilemma of choice that conforms to Special Relativity but is still recognisably consistent with conventional probability theory.

Note that even though both expressions *p*(*A* or *B*|*C*) and *p*(*A* and *B*|*C*) commute for *A* and *B*, Bayes’ Theorem *does* distinguish the precedence of the conditionalities associated with the primary events.

Thus, Equation (2b) can be expressed in one of two ways, according to which of the primary events *A* and *B* is prior: substituting Equation (2c) into Equation (2b), we find two choices offering two possibilities for the logical OR function:

*p*(*A* or *B*|*C*) = *p*(*A*|*C*) + *p*(*B*|*C*) − *p*(*A*|*BC*) *p*(*B*|*C*)       = *p*(*A*|*C*) + *p*(*B*|*C*) − *p*(*B*|*AC*) *p*(*A*|*C*) (2d)

Equation (2d) may be simplified by noting that the component term of Bayes’ Theorem appearing at the end of each CSR expression (each such latter term representing a conditional possibility) can also be assigned a probability associated with the truth of that conditionality. This allows us to informally re-write Equation (2d) as a single statement, by expanding upon the products from Equation (2c):

*p*(*A* or *B*|*C*) = *p*(*A*|*C*) + *p*(*B*|*C*) − {[*p*(*A*|*BC*) *p*(*B*|*C*)]*_b_* + [*p*(*B*|*AC*) *p*(*A*|*C*)]*_a_*} (5a)

where the subscripts {*a*, *b*} appearing after each set of square bracket expressions informally indicate the assumed prior for each square bracket expression. The expression in each of the pair of square brackets represents the respective Bayes’ Theorem choice. Equation (5a) can be regularised by including the probability associated with the prior, that is, now explicitly including a recursive element. That is to say, in interpreting the subscripts {*a*,*b*} of Equation (5a), say ‘*a*’ (on the RHS) then this (informally) indicates the probability associated with the event *A* being true and having occurred. So, one might perhaps, in the first instance, simply want to multiply again by the probability of *A*, *p*(*A*|*C*). However, we are entertaining here the two ideas of conditionality *and* recursiveness; in which case employing the conditional probability *p*(*A*|*BC*), instead of *p*(*A*|*C*), recognises the ongoing conditionality of *A* on *B*, so that the inclusion of *p*(*A*|*BC*) therefore also builds in a desired recursive aspect into Equation (5a). The same reasoning is used *mutatis mutandis* for the ‘*b*’ subscript.

Note that Equation (5a) requires that {*A*,*B*} are *distinct*, that is, the possibility *A* = *B* is excluded. (But note that Equation (8) is also valid for the case *A* = *B*. This is proved formally: see Equation (12).)

Note also that the probability *p*(*A*|*BC*) associated with the prior is *not* the same as the probability of the prior, which we may state informally as: *p*(“*A* is prior to *B*”). Thus, we explicitly include the probabilities associated with the priors (removing the subscripts {*a*,*b*}). In effect, we now assume the possibility of a ‘prior prior’ and the (logical/temporal) ordering of the events {*A*,*B*} now also starts to be explicitly taken into account:

*p*(*A* or *B*|*C*) = *p*(*A*|*C*) + *p*(*B*|*C*) − {[*p*(*A*|*BC*) *p*(*B*|*C*)] *p*(*B*|*AC*) + [*p*(*B*|*AC*) *p*(*A*|*C*)] *p*(*A*|*BC*)} (5b)

allowing a consistent (and symmetric) simplification which intrinsically includes the possibility of recursion:

*p*(*A* or *B*|*C*) = {*p*(*A*|*C*) + *p*(*B*|*C*)} {1 − *p*(*A*|*BC*) *p*(*B*|*AC*)} (5c)

In Equation (5c) we have the probability of “*A* or *B* given *C*“ being {[the probability of *A* given *C*] or [the probability of *B* given *C*]} reduced by a cross-probability factor *F*, where

*F* ≡ −*p*(*A*|*BC*) *p*(*B*|*AC*) (6a)

which could be thought to represent a “first level” of dependency, that is, the dependence of *A* on *B* (and vice versa). But what if we also consider a deeper dependence between these events? This would give the following:

*p*(*A* or *B*|*C*) = {*p*(*A*|*C*) + *p*(*B*|*C*)}{1 + *F* + *F*^2^ + …)} (6b)

or, continuing until *dependence* becomes *recursion*, as follows:

(6c) $$p\left(\left.A\;OR\;B\right|C\right)=\left\{p\left(\left.A\right|C\right)+p\left(\left.B\right|C\right)\right\}\sum_{m=1}^{\infty }\left\{1+{\left[-p\left(\left.A\right|BC\right)p\left(\left.B\right|AC\right)\right]}^{m}\right\}$$

Given that probabilities may not be greater than unity this infinite series can be compactly written as follows:

(7) $$p\left(\left.A\;OR\;B\right|C\right)=\frac{p\left(\left.A\right|C\right)+p\left(\left.B\right|C\right)}{1+p\left(\left.A\right|BC\right)p\left(\left.B\right|AC\right)}$$

However, Equation (7) may be simplified since, given that in the recursive case it cannot be determined which of {*A*,*B*} is prior, we may take *p*(*A*|*B*) = *p*(*A*) and *p*(*B*|*A*) = *p*(*B*). Hence:

(8) $$p\left(\left.A\;OR\;B\right|C\right)=\frac{p\left(\left.A\right|C\right)+p\left(\left.B\right|C\right)}{1+p\left(\left.A\right|C\right)p\left(\left.B\right|C\right)}$$

Equation (8) (isomorphic to Equations (1)) states the “Hyperbolic Sum Rule” (HSR) theorem to be proved where {*A*,*B*} are recursively dependent. In deriving the form of Equation (8), we asserted that *p*(*A*|*BC*) = *p*(*A*|*C*) and *p*(*B*|*AC*) = *p*(*B*|*C*); that is, due to the recursion between events {*A*, *B*}, the probability of *A* becomes essentially independent of *B*, and vice versa. That is, Equation (7) can be re-expressed in the strictly hyperbolic form of Equation (8). In effect, this assertion (*p*(*A*|*B*) = *p*(*A*), etc.) can be justified as the most likely (MaxEnt) relationships between the conditional probabilities for *A* and *B* when we know a priori that *A* and *B* may have a recursive dependency, but we do not know the precise (quantitative) relationship between *A* and *B*, or indeed, the priority of *A* or *B*. In Appendix A.3 we prove that the HSR of Equation (8) is MaxEnt. That is to say, these assertions can also be justified by recourse to the *Principle of Indifference* (PI) which is the simplest non-informative prior in Bayesian probability (see [36,38]).

Equation (6b) expresses the case where the relation between events {*A*, *B*} is not recursive but stops at a countable depth or iteration: the overt conditionality between them is therefore maintained such that *p*(*A*|*B*) ≠ *p*(*A*) and *p*(*B*|*A*) ≠ *p*(*B*). For example, if one wanted to formalise the psychological aspects of Poker, one might express a “bluff” as the *F* term in Equation (6b), a “double-bluff” as the *F*^2^ term, and a “triple-bluff” as the *F*^3^ term etc. But the HSR of Equation (8) expresses the recursion needed for the etalon of Equation (1c) (for example). In contrast, it is clear that the CSR of Equation (2b) is the truncated HSR case of Equation (7), where the CSR a priori ignores recursion and its associated higher-order dependencies (either due to specified information, or because their existence is simply not taken into account) and excludes the higher-order (logical/temporal) ordering of propositions. In any case, Bayes’ Theorem (the Product Rule) remains valid.

### 2.4. The Conventional Sum Rule (II)

Both the HSR (Equation (8)) and the CSR (Equation (2b)) are MaxEnt in each of their respective domains of applicability (proved, respectively, in Appendix A.3 and Appendix A.5), and we need to know when to use the CSR and when the HSR (and why). In particular, the point of this paper is to emphasise the importance of the temporal (or logical) *ordering* of propositions, and therefore the possibility for recursion, which is what distinguishes between the CSR and HSR: the CSR does not adequately take the temporal ordering of propositions into account, whereas the HSR does. And, this is particularly evident in the Venn diagram of Figure 1 which cannot represent the temporal or logical ordering of the propositions.

As an example, we consider the scenario of a fair coin being tossed twice. We designate the event *A* as “the first toss gives a head”, and the event *B* as “the second toss gives a head”, and we wish to compute the probability of the first toss *or* the second toss giving a head. Such a ‘fair’ coin does not have memory (each toss is independent of any others), so that we have *p*(*A*) = *p*(*B*) = ½. Here, there is no recursion since *p*(*C*|*A*) = *p*(*C*) and *p*(*C*|*B*) = *p*(*C*): that is, *C* is independent of both of {*A*,*B*}, and therefore we may simply use the CSR (Equation (2a)): *p*(*A* or *B*|*C*) = ½ + ½ − ¼ = ¾.

Clearly, applying the HSR (Equation (8)) to obtain *p*(*A* or *B*|*C*) = 4/5 in this context is manifestly false, precisely because *we already know* that there is no recursion present. The difference between the hyperbolic (HSR, Equation (8)) and conventional (CSR, Equation (2b)) sum rules is specifically the *prior information* that must be taken into account to do the calculation. The decisive question is whether or not the possibility that the mutual dependence of {*A*,*B*} is recursive can be excluded.

There are other simple cases where memory is involved, such that *p*(*A*|*B*) ≠ *p*(*A*). One such case is Cox’s [1] example of randomly drawing black or white balls from a bag. The probability of a result is affected by the results of previous draws, but this is a *memory* effect, not a *recursive* one; and here the system only iterates based on the forward propagation of a previous state (i.e., no feedback): see Appendix A.6 for a discussion of FIR (finite impulse response) filters. In general, if *p*(*A*|*B*) and *p*(*B*|*A*) are expressed in non-recursive form the CSR should be used, but if not, then the HSR should be used. The CSR should be used only where recursion is a priori excluded.

## 3. A General “Hyperbolic Sum Rule” (HSR)

### 3.1. Proving the HSR Theorem

We seek to prove the “Hyperbolic Sum Rule” of Equation (8) (whose plausibility was described informally in Section 2.3):

$$p\left(\left.A\;OR\;B\right|C\right)=\frac{p\left(\left.A\right|C\right)+p\left(\left.B\right|C\right)}{1+p\left(\left.A\right|C\right)p\left(\left.B\right|C\right)}$$

with the notation defined in Section 2. Consider the function Σ given previously as Equation (4a):

$$\Sigma \left(x\right)=\frac{1-x}{1+x}$$

which has the same properties as Cox’s function *S*, such that *v* ≡ Σ(*u*), where *u* ≡ *p*(*A*|*C*) and therefore *v* ≡ *p*($\bar{A}$|*C*), properties noted by both Jaynes [2] and Cox [1]. Also Σ{Σ(*x*)} = *x* just as *S*{*S*(*x*)} = *x*.

From de Morgan’s Theorem ($\bar{A\;OR\;B}\equiv \bar{A}\bar{B}$, hence $A\;OR\;B\equiv \bar{\bar{A}\bar{B}}$), and see also Equation (13) *passim* in Cox [1]:

(9) $$p\left(\left.A\;OR\;B\right|C\right)=\Sigma \left[\Sigma \left(p\left(\left.A\right|\bar{B}C\right)\right)\Sigma \left(p\left(\left.B\right|C\right)\right)\right]$$

where $\bar{B}$ (the negation of *B*) is required in the conditioning probability of the first probability term in order to satisfy the appropriate application of the Product Rule in de Morgan’s Theorem.

Substituting Cox’s *first-order expression* for Σ (that is, Σ = 1 − *x*) we recover the “Conventional Sum Rule” (CSR, Equation (2b)), after some manipulation. However, by substituting the full bilinear form for Σ (Equation (4a)) into Equation (9):

(10) $$\begin{array}{ll} p\left(\left.A\;OR\;B\right|C\right) & =\Sigma \left[\frac{\left(1-p\left(\left.A\right|\bar{B}C\right)\right)\left(1-p\left(\left.B\right|C\right)\right)}{\left(1+p\left(\left.A\right|\bar{B}C\right)\right)\left(1+p\left(\left.B\right|C\right)\right)}\right]\\ & =\Sigma \left[\frac{1-p\left(\left.A\right|\bar{B}C\right)-p\left(\left.B\right|C\right)+p\left(\left.A\right|\bar{B}C\right)p\left(\left.B\right|C\right)}{\left(1+p\left(\left.A\right|\bar{B}C\right)\right)\left(1+p\left(\left.B\right|C\right)\right)}\right] \end{array}$$

and expanding the outer self-reciprocal Σ function we find:

(11) $$\begin{array}{ll} p\left(\left.A\;OR\;B\right|C\right) & =\frac{1-\left(\frac{1-p\left(\left.A\right|\bar{B}C\right)-p\left(\left.B\right|C\right)+p\left(\left.A\right|\bar{B}C\right)p\left(\left.B\right|C\right)}{\left(1+p\left(\left.A\right|\bar{B}C\right)\right)\left(1+p\left(\left.B\right|C\right)\right)}\right)}{1+\left(\frac{1-p\left(\left.A\right|\bar{B}C\right)-p\left(\left.B\right|C\right)+p\left(\left.A\right|\bar{B}C\right)p\left(\left.B\right|C\right)}{\left(1+p\left(\left.A\right|\bar{B}C\right)\right)\left(1+p\left(\left.B\right|C\right)\right)}\right)}\\ & =\frac{2p\left(\left.A\right|\bar{B}C\right)+2p\left(\left.B\right|C\right)}{2+2p\left(\left.A\right|\bar{B}C\right)p\left(\left.B\right|C\right)} \end{array}$$

But, when *A* depends recursively on *B*, then also *B* always depends on *A*, so that *p*(*A*|*B*) = *p*(*A*|*A*); hence *p*(*A*|*B*) = *p*(*A*), also *p*(*A*|$\bar{B}$) = *p*(*A*); and therefore $p\left(\left.A\right|\bar{B}C\right)=p\left(\left.A\right|C\right)$.

Thus:

(12) $$p\left(\left.A\;OR\;B\right|C\right)=\frac{p\left(\left.A\right|C\right)+p\left(\left.B\right|C\right)}{1+p\left(\left.A\right|C\right)p\left(\left.B\right|C\right)}$$

which is Equation (8) as required. Note that *p*(*A*|*B*) = *p*(*A*) if {*A*,*B*} are *either* independent (a CSR case) *or* recursive (the HSR case).

### 3.2. Some Analytical and Numerical Comparisons

Having derived a “Hyperbolic Sum Rule” (HSR) expression, it is interesting to see how much it diverges from the “Conventional Sum Rule” (CSR) in a simplified scenario that considers the function *p*(*A* or *B*) for two possible and equally probable events {*A*, *B*}, that is, *p*(*A*) = *p*(*B*).

The HSR is well-behaved in that it yields the same results as the CSR in the limit that neither event occurs, that is, *p*(*A*) = 0 and *p*(*B*) = 0. In this case, we have *p*(*A*|*BC*) = *p*(*B*|*AC*) = 0 (that is, *p*(*A* and *B*|*C*) = 0 from Bayes’ Theorem). It is straightforward to see in this specific case that *p*(*A* or *B*) = 0 for both sum rules.

The HSR is also well-behaved where *p*(*A*) = *p*(*B*) = 1. In this case, the CSR reduces to *p*(*A* or *B*) = *p*(*A*) + *p*(*B*) − *p*(*B*) = *p*(*A*) = *p*(*B*). The HSR also reduces to the same result, but via the progression *p*(*A* or *B*) = 2*p*(*A*)/2 = 2*p*(*B*)/2 = *p*(*A*) = *p*(*B*).

**Figure 2:**
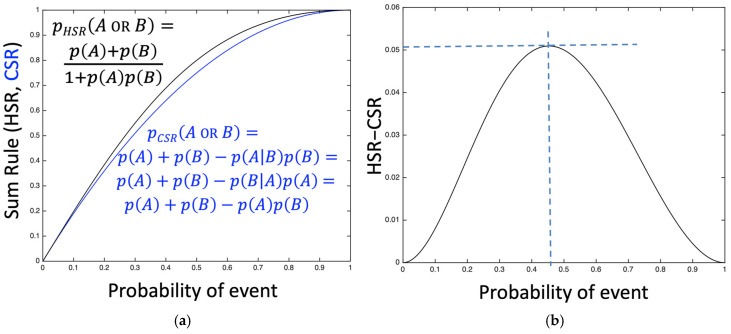
Comparing the HSR and CSR versions of *p*(*A* or *B*) for two equally probable hypotheses {*A*,*B*}, where {*A*,*B*} are assumed independent for the CSR case. (**a**) Hyperbolic Sum Rule (HSR: black) and Conventional Sum Rule (CSR: blue); (**b**) Difference between HSR and CSR.

For the simple system in which there are two equally probable events {*A*,*B*} the conventional and hyperbolic forms of the sum rule converge when the events are either impossible or certain. It is for the intermediate probabilities 0 < *p*(*A*) < 1 that the two forms diverge. Figure 2 shows the behaviour of *p*(*A* or *B*); calculated for the simple case of *p*(*A*) = *p*(*B*). For the CSR the two events {*A*,*B*} are assumed to be independent, that is, *p*(*A*|*B*) = *p*(*A*) and *p*(*B*|*A*) = *p*(*B*), according to the MaxEnt criterion (in the absence of any other information).

From a simplistic Maximum Entropy (MaxEnt) argument, one might expect that the greatest difference between the two sum rules would occur when the events have the most uncertainty, that is, *p*(*A*) = ½. For this situation, the CSR returns an overall probability of *p_CSR_*(*A* or *B*|*C*) = ½ + ½ − (½ × ½) = ¾ (75%). In contrast, the HSR returns a slightly higher overall probability of *p_HSR_*(*A* or *B*|*C*) = (½ + ½)/(1 + [½ × ½]) = ⅘ (80%). The presence of recursion (or mutual dependence) *increases* the probability that both events will occur.

Interestingly, however, Figure 2 shows that the maximum difference between the two sum rules is actually 5.09 percentage points, for *p* = 45.35% in this simplified case (reflecting the asymmetry of the hyperbolic tangent function). Thus, we see that the difference between the conventional and hyperbolic sum rules may be quite minor: in most cases it would be hard to observe (less than 5 percentage points).

Being MaxEnt (proved in Appendix A.3), an application of the HSR of Equation (12) would be to calculate the sum probability for two events where the relationship between the two events is not known, yet some correlation (including recursion) is reasonably anticipated or cannot be excluded. In contrast, the CSR (also MaxEnt, proved in Appendix A.5.2) explicitly assumes that recursion is not present and also requires knowledge of the conditional probability relationship between the two events: this may be estimated or simply assumed not to exist (since independence is a reasonable MaxEnt assumption). Note that the CSR explicitly excludes recursion, an assumption which should be justified. Thus, the HSR of Equation (12) obviates both the need for any unknown conditional probability, and also avoids any unjustified assumptions relating to recursion.

For example, imagine a scenario of elections occurring on the same future day in neighbouring countries “A” and “B”, with leading candidates “Alice” and “Bob” in each, respectively. Opinion polls consistently suggest that both candidates have a good chance of being elected: 45% in each case: *p*(*A*) = *p*(*B*) = 0.45. We pose the question (to which we would like a Bayesian answer, if possible): what is the probability that at least one of the two leading candidates will be elected?

In order to apply the conventional sum rule we require the conditional probability that Alice will be elected given Bob is elected, or alternatively, the probability that Bob is elected given Alice is successful (given by Bayes’ Theorem, Equation (2c)). In the CSR context (in the absence of any other information to the contrary) the best we can assume is that these events are essentially independent, in which case *p*(*A*|*B*) = *p*(*A*).

The CSR can then be applied to offer the MaxEnt probability of either Alice or Bob being elected (given that the two events are mutually independent):

*p*(*A* or *B*)_CSR_ = *p*(*A*) + *p*(*B*) − *p*(*A*)*p*(*B*) = 0.698

However, the HSR allows us to include the possibility of a mutual (recursive) relationship between these two events (without making any other assumptions which could skew the calculation), giving a MaxEnt (most likely) probability in the absence of any other information (or any implicit assumptions):

*p*(*A* or *B*)_HSR_ = {*p*(*A*) + *p*(*B*)}/{1 + *p*(*A*)*p*(*B*)} = 0.748

Thus, the HSR probability estimation of at least one of the candidates being successful is noticeably higher than that anticipated from the CSR. This is because there are a variety of ways the two elections could potentially influence each other, and the HSR accounts for the possible existence of these mutual recursive interactions, while (in the absence of specific information as given by the appropriate conditional probabilities) the CSR explicitly ignores such possible recursive influences.

It is perhaps surprising that such a difference between the two sum rules has not been noted up to now: surely empirical observation would have shown a systematic discrepancy from the (wrong) current theoretical calculation based purely on the CSR? That no-one has suspected that the CSR is faulty may be due to the fact that for most simple cases (e.g., card games, gambling etc.) recursion is generally absent (and the CSR is correct). Anyway, probability theory originated in the analysis of games of chance where recursion is excluded. For more complex cases there is usually no practical possibility of re-running the observations (elections in the case given above) sufficiently to gain a statistically significant estimate of the associated empirical probabilities. Most real cases have complex (and recursive) conditionalities and dependencies, and most are unrepeatable, meaning that the associated probabilities cannot be determined by conventional (frequentist) methods. Thus, any systematic error in the underlying probability theory is probably unobservable, and it is hardly surprising that it has been unobserved and unsuspected until now.

### 3.3. Concatenation Rules for Multiple Hypotheses

In Appendix A.4 we prove the following relations:

A version of Bayes Theorem for multiple summed hypotheses {*X*}_OR_ as the number of these hypotheses grows large (see Appendix A.4 Equation (A18)):

$$\begin{array}{c} p\left(A\right)p\left(\left.{\left\{X\right\}}_{\mathrm{OR}}\right|A\right)=p\left({\left\{X\right\}}_{\mathrm{OR}}\right)p\left(\left.A\right|{\left\{X\right\}}_{\mathrm{OR}}\right)\;as\;\left\{X\right\}\to \infty \end{array}$$

We also exploit the hyperbolic tangent function to prove a Hyperbolic Sum Rule (HSR) for multiple hypotheses with multiple conditionalities (see Appendix A.4 Equation (A24)):

$$p{\left(\left.A\;OR\;B\right|D\;OR\;E\right)}_{\mathrm{HSR}}=\frac{p\left(\left.A\right|D\;OR\;E\right)+p\left(\left.B\right|D\;OR\;E\right)}{1+p\left(\left.A\right|B\;OR\;D\;OR\;E\right)p\left(\left.B\right|A\;OR\;D\;OR\;E\right)}$$

Finally, a generalised HSR with *N* multiple hypotheses, which sorts odd-ordered conjunctions into the numerator and even-ordered conjunctions into the denominator (see Appendix A.4 Equation (A25)):

$$p{\left(A_{1}\;OR\;A_{2}\;OR\;\ldots \;OR\;A_{N}\right)}_{\mathrm{HSR}}=\frac{\sum_{l=1,\mathrm{odd}}^{N}\sum_{m=1}^{m=\frac{N!}{l!\left(N-l\right)!}}\prod_{n=P\left\{l,1:N\right\}}^{l}p\left(\left.A_{n}\right|\left\{A_{n^{\prime }},l-1\right\}\right)}{\sum_{l=0,\mathrm{even}}^{N}\sum_{m=1}^{m=\frac{N!}{l!\left(N-l\right)!}}\prod_{n=P\left\{l,1:N\right\}}^{l}p\left(\left.A_{n}\right|\left\{A_{n^{\prime }},l-1\right\}\right)}$$

We note the close functional isomorphism of Equation (A25) with the transfer function (defined by the *Z*-transform) of an infinite impulse response (IIR) filter used in digital signal processing (see further in Appendix A.6), which is given by a quotient of summations in both the numerator and denominator:

(13) $$Z\left(z\right)=\frac{\sum_{l=0}^{L}b_{l}z^{-l}}{1+\sum_{n=1}^{N}a_{n}z^{-n}}$$

The parameter *z*, is given by *z* ≡ exp(i*ω*), where *ω* is a frequency in normalised units, such that *z* is therefore located on the unit circle in the complex plane (see the discussion in Section 2.2 (Equation (4c)) for the self-reciprocal function *S^m^*, whose roots also lie on the unit circle), noting that for convenience the coefficients *a_n_* in Equation (13) are represented negatively as compared to convention. Of particular interest is to note that the conventional IIR filter generally has the first co-efficient of the denominator as unity, just like the HSR of Equation (12).

Equation (A25) may also be written in a form such that the probability distributions are represented (as MaxEnt decaying exponentials probabilities, see Jaynes 1982 [3]) by the appropriate Lagrange multiplier *β* (noting that different distributions are generated by different choices of *β*, with an example of this given in a recent treatment of the Wine/Water paradox [38]) (see Appendix A.5 Equation (A28d)):

$$p{\left(A_{1}\;OR\;A_{2}\;OR\;\ldots \;OR\;A_{N}\right)}_{\mathrm{HSR}}=\frac{\sum_{l=1,\mathrm{odd}}^{N}\frac{N!}{l!\left(N-l\right)!}{\left\{\left(\frac{1-e^{-\beta }}{1-e^{-N\beta }}\right)e^{\beta }\right\}}^{l}exp\left\{-\beta \sum_{n=P\left\{l,1:N\right\}}^{l}n\right\}}{\sum_{l=0,\mathrm{even}}^{N}\frac{N!}{l!\left(N-l\right)!}{\left\{\left(\frac{1-e^{-\beta }}{1-e^{-N\beta }}\right)e^{\beta }\right\}}^{l}exp\left\{-\beta \sum_{n=P\left\{l,1:N\right\}}^{l}n\right\}}$$

additionally showing that the HSR is well-behaved for multiple hypotheses as N→∞.

In Appendix A.5 we prove the following relation for the Conventional Sum Rule for multiple hypotheses (see Appendix A.5.1 Equation (A34)):

$$\begin{array}{c} p{\left(A_{1}\;OR\;A_{2}\;OR\;\ldots \;OR\;A_{N}\right)}_{\mathrm{CSR}}=\\ 1+\sum_{l=1,odd}^{N}\sum_{m=1}^{m=\frac{N!}{l!\left(N-l\right)!}}\prod_{n=P\left\{l,1:N\right\}}^{l}p\left(\left.A_{n}\right|\left\{A_{n^{\prime }},l-1\right\}\right)-\sum_{l=0,even}^{N}\sum_{m=1}^{m=\frac{N!}{l!\left(N-l\right)!}}\prod_{n=P\left\{l,1:N\right\}}^{l}p\left(\left.A_{n}\right|\left\{A_{n^{\prime }},l-1\right\}\right) \end{array}$$

This relation for the CSR (Equation (A34)) rests on results depending on the HSR.

## 4. Discussion

### 4.1. Probability Is Physical

In most cases, the distinction between the Conventional Sum Rule (CSR) and the Hyperbolic Sum Rule (HSR) is probably unobservable, given the attainable empirical precision. The case of the two parallel elections considered in Section 3.2 has 80% for the HSR compared to only 75% for the CSR, when the two election probabilities involved are both 50% (*p*(*A*) = *p*(*B*) = 0.5). But the CSR cannot model recursion.

However, the derivation of the HSR uncovers a mathematical relation that is also observed in diverse physical settings. In particular, the HSR is isomorphic not only to the hyperbolic tangent double-angle identity (Equation (1a)) but also to Einstein’s famous velocity addition formula (Equation (1b)) and to the reflectivity expression for an optical etalon (Equation (1c)). Thus, the general (hyperbolic) probability sum rule conforms to a ‘template’ seen in the physical world; that is to say, although being apparently a *logical* relation it behaves just as many physical phenomena do. Therefore, we can say that the etalon function (Equation (1c)): intrinsically probabilistic in nature, being a wave scattering phenomenon) represents at least a *physical embodiment* of the HSR. In particular, the physical structure of the etalon with its two scattering interfaces (*A* and *B*, say) suggests that the reflection coefficient calculated from the solution to Equation (1c) can also be interpreted as representing the physico-logical answer to the question: what is the probability that a quantum-mechanical particle (that is, a photon) is reflected from interfaces “*A* OR *B*”? It is clear that the basic two-faceted etalon physically expresses the logical OR operation.

This raises the interesting philosophical question: is the hyperbolic nature of the HSR a result of the intrinsic hyperbolic character of the natural universe? Affirming that it is implies that probability theory is firmly empirical (and not merely mathematical): both the theory of Special Relativity (Equation (1b)) and the quantum-mechanical scattering probability (Equation (1c)) are consistent with the hyperbolic nature of spacetime. But denying it would imply that it is simply *coincidental* that the HSR shares fundamental characteristics of the natural universe.

This question actually goes to the root of a deep philosophical debate as to the intrinsic nature of probability theory: is it purely an empirical construct where probabilistic variables only acquire meaning when a statistical measurement takes place? This view is implied by the so called ‘frequentist’ fraternity, who do not ascribe an independent reality to the “laws” of probability; rather they consider that the probability of a hypothesis depends solely upon the frequency of success over a given number of trials. But Bayesians would say that the hyperbolic nature of the HSR implies that probability theory and the underlying theoretical equations form a component part of the full set of universal natural laws; that is to say, they are an intrinsic aspect of the (hyperbolic) universe with their own independent existence.

We should add that the result that the MaxEnt nature of both the HSR of Equation (12) (possibly recursive, see Appendix A.3) and the CSR (non-recursive, see Appendix A.5) indicates that the *equilibrium* state of physical systems (corresponding to a stable and most-likely configuration) obeys both the *kinematical* Principle of Least Action and the *entropic* Principle of Least Exertion ([11]). These fundamental principles underlie all known physical phenomena and therefore indicate that both the HSR and CSR are consistent with the requirements of the physical world. The fact that the HSR is MaxEnt also implies that all the parts of such systems recursively condition all the other parts. That is, in the general case everything is entangled with everything else just as Karen Barad implies [34]. But here we wish to avoid metaphysical discussion (for which see [6,7]).

### 4.2. Recursion

Why has the issue of recursion apparently not previously been explicitly considered in probability theory? This may simply be an accidental result of history, with probability theory having developed from the gaming scenarios associated with cards and dice, where events are discretely independent and are characterised with shallow conditionalities that do not reach far back into the past. For example, the game of Poker has explicit calculable conditionalities based on cards revealed to the table, cards in your hand, and the hidden cards remaining; the calculable conditionalities do not involve the (fascinating) issues of bluffing, double bluffing, and multiple bluffing. These could in principle be expressed logically (using the formalism sketched above), but are currently treated as psychology.

However, it is interesting to note that Cox (1946 [1]) derives the general result *S^m^* = 1 − *x^m^* for the involutory function *S*(*x*), stating that the value for *m* (assumed an integer) is “*arbitrary*” and “*purely conventional*”; indeed, Jaynes writes that the value of *m* is “*actually irrelevant*” [2]. For “*simplicity of notation*” Cox arbitrarily chooses *m* = 1 (to effectively define Equation (3c)), which is both convenient and intuitive, since it immediately leads to the Conventional Sum Rule associated with the Venn diagram of Figure 1. Thus, Cox’s analysis allows for recursion conceptually, although he apparently appreciated neither this nor the physical interpretation of his function *S^m^*.

But exploiting the hyperbolic insight described here, we can now understand *m* to represent the degree of mutual dependency leading to recursion; that is, it has both a logical and physical (spatio-temporal) meaning, with real physical implications which are not at all “arbitrary”. In particular, *m* = 0 is trivial, *m* = 1 is the simple CSR case, *m*^−1^ = 0 is the HSR case. We consider this further in Section 4.3.

Another reason for ignoring recursion is that the appropriate calculus of probabilities requires substantial computing power which has only become available in recent decades. Up to the point of the emergence of artificial intelligence (AI) and machine learning (ML), approximately at the turn of the millennium, there has not been a good reason to develop a theory of recursive probabilities, since before this time the necessary computations were practically intractable. However, as large-scale computing power has evolved over recent decades, this has also enabled the development of the sophisticated (Bayesian) algorithms underpinning AI and ML programming. Such algorithms allow a dispassionate approach to making probabilistic inferences and decision-making in an environment of uncertainty, within a mathematical framework for updating beliefs and reasoning about uncertain events. In addition, recursion provides AI with additional flexibility to handle complex and dynamic problems by recursively decomposing them, while leveraging self-referential architectures and utilising iterative processes.

Recursion can be exploited relatively simply in AI as a means to break down complex problems into smaller, more manageable subproblems. Alternatively, the complexity and power of modern computing also enables the employment of recursive data structures that can be used to represent hierarchical or interconnected relationships. Recursion can also be seen in the inductive reasoning aspect of AI, which involves inferring general rules or patterns from specific observations, so as to learn iteratively from data in order to improve the accuracy of predictions or classifications.

In AI (and the associated field of neural networking) recursion has been employed empirically: the complexity associated with the algorithms and feed-back routines are not generally amenable to analytical (closed-form or tractable mathematical) analysis, such that currently only ‘simulations’ can be used to analyse AI behaviour. It is in this context of recursive AI that the Hyperbolic Sum Rule described here allows the systematic, reliable and consistent handling of multiple recursive hypotheses (or events) according to Bayesian requirements, as discussed in Appendix A.4. We therefore expect the HSR to enable a more analytic (closed-form and, therefore, tractable) analysis of complex AI algorithms to give additional insights into their form of operation and anticipated outputs.

### 4.3. Two Distinct Sum Rules

We have shown that in different situations different rules apply for summing the probabilities of events (or, nearly equivalently, the probabilities of certain hypotheses being true). We wish to find a relation that allows us to compute *p*(*A* or *B*|*C*), that is, the probability of (*A* or *B*) given *C*, where {*A*,*B*} are events (or hypotheses) and {*C*} are conditionalities.

There are two distinct cases depending on whether or not {*A*,*B*} are recursively dependent. If {*A*,*B*} are independent (such as coin flipping, see Section 2.4) or they are not recursively dependent then the “*Conventional Sum Rule*” (**CSR**) applies. But if {*A*,*B*} are recursively dependent (such as reflection from a multilayer stack, see Equation (1c)) or indeed, if their mutual relationship is not quantitively known (such that recursion cannot be ruled out) then the “*Hyperbolic Sum Rule*” (**HSR**) applies. Concatenation formulae for either or both of multiple events {*A*,*B*, …} or multiple conditionalities {*C*,*D*, …} are readily derived for both the HSR (Appendix A.4), and the CSR (Appendix A.5; also see the summary in Section 3.3 of important results for both HSR and CSR). Therefore, there is no loss of generality from treating only the simple cases.

If *p*(*A*|*B*) > 0 and *p*(*B*|*A*) > 0, with both conditional probabilities being valid independent of any temporal (that is, causal) or logical constraints, then {*A*,*B*} are recursively dependent and the HSR applies. Otherwise, the CSR applies. Conversely, the CSR applies if this probability relation holds:

[{*p*(*C*|*A*) = *p*(*C*) or *p*(*C*|*B*)} = *p*(*C*)] = {*p*(*C*|*A* or *B*) = *p*(*C*)}

Note that in simple cases (see Figure 2) the difference between CSR and HSR is probably too small to be easily observed: even in the fair coin or election cases (Section 2.4 and Section 3.2) the CSR gives 75% where the HSR gives 80%.

It is clear that the CSR and HSR are formally related (see the discussion in Appendix A.5.2 and Equation (4)). It also seems fairly clear that the “truncated expansion” of Equation (4d) has properties related to (for example) the “finite impulse response” (“FIR”) filter that is important in digital signal processing (DSP) applications (further details in Appendix A.6). The case represented by finite *m* where *m* ≥ 1 and *m*^−1^ > 0 (analogous to the FIR filter) offers an engineering approach to the probabilistic treatment and control of system noise within a finite (and specified) timeframe, an issue of practical importance for stochastic systems. Such practical probabilistic cases, formally CSR (see Appendix A.5), blur the distinction between CSR and HSR, since it seems that such “FIR filtering” may be represented approximately as a “truncated HSR”, with a formalism that is also approximately MaxEnt in its own terms (see Appendix A.3). However, the derivation of the HSR is directly from Equation (4a): the discussion of finite values of *m* in Equation (4b,c,d) is presented only by way of comment. The DSP engineers have the “infinite impulse response” (“IIR”) filter, which in our representation is isomorphic to the HSR, but the HSR is defined without any (infinite) summations. There is the (possibly) recursive case (HSR) and the (definitely) non-recursive case (CSR).

### 4.4. Motivation for the HSR: An Example

The artificial intelligence (**AI**) and machine learning (**ML**) applications widely employed today regularly use Bayesian methods to address complex problems involving multiple hypotheses each potentially featuring multiple conditionalities. One such problem is the non-trivial one of how to build a Battery Management System for the electric vehicle (EV) sector, seeking to optimise longevity and lifecycle profile, distance between recharging, recyclability, and overall environmental impact; even providing guidance for driving style to help achieve such an optimisation, in advance of the availability of full automotive self-driving technology. This has attracted significant interest (see, for example, Ghalkhani and Habibi [45]), and is supported in a current EU Horizon programme [46], CIRCUBATT: Circular economy innovations for resilient, competitive and sustainable battery technologies.

A battery pack appears to be a simple thing, but there is much complexity associated with it, as well as its optimised exploitation, and safeguarding measures to minimise risk since such intensive energy storage capability remains hazardous. The CIRCUBATT research programme, which aims to develop commercially viable applications, generates very large datasets based on a multitude of space- and time-stamped parameters generated by the dynamic properties of a large base of EVs and their batteries, whose analysis is facilitated by AI/ML tools which rely crucially on recursion as a means to achieve efficient learning, optimisation and decision-making processes.

The architecture of an AI/ML system using Bayesian inference within its processing engine consists of multiple layers (e.g., an input layer, which receives the input data, hidden layers which process the input data, and an output layer), and frequently features recursion as a means to achieve convergence of the output: recursion being exhibited when an algorithm calls a function or process repeatedly until convergence is achieved. Each layer of the AI/ML system receives multiple inputs, which can be considered to be the prior probabilities (hypotheses or initial beliefs), and outputs into the inputs of the next layer. For example, at an intermediate plane, the various states input into that layer can each represent the possibility of hypotheses (A or B or C or …) considered in the intermediate calculation, where “or” here indicates the Sum Rule to effect the summation of the hypotheses: conventionally the Sum Rule would have been the CSR but now the HSR has been shown to be the correct MaxEnt rule to use; particularly in the absence of a definitive set of a priori rules or contextual constraints (i.e., apart from any known initial learning parameters) for the AI/ML learning phase. Having been fed forward through a number of intermediate stages, the AI algorithm often features recursion by feeding the output results back into its input, and repeating the whole process, before converging to a final result.

Analysing such an AI/ML process analytically is practically impossible when the CSR is employed, as indeed is guaranteeing that the calculation will be ‘well behaved’, or is indeed ‘reliable’, for example when AI/ML is used in a safety-critical context such as autonomous vehicles. Currently, in effect, AI calculations can only be analysed by simulation to see what the output will be. A priori prediction of the result of the AI calculation is effectively impossible currently, as is guaranteeing its reliability and safety, or indeed ensuring a degree of explication and predictability. But concatenation of the HSR, and indeed, ‘nesting’ of HSR expressions within a HSR function, in order to analytically model such a multi-stage and recursive AI architecture is in principle both analysable and tractable. This therefore provides a route to the analytical study and assured understanding of complex AI/ML processes and calculations.

Particularly for probabilistic AI/ML schemes, sigmoid or softmax functions are often used for the non-linearity frequently required in the intermediate (hidden) stages to achieve probabilities and classifications, and it is noteworthy that the HSR automatically embodies such a non-linear (*tanh*) function, thereby already making it inherently suitable for use in such AI/ML processing; again, the MaxEnt feature of the HSR and the *tanh* function (see Appendix A.3) is also relevant here with the minimal introduction of unwanted, undesired, or unintended extraneous information by the non-linear function into the final solution.

## 5. Summary and Future Work

We have shown how a *Hyperbolic Sum Rule* (**HSR**) for probability can be derived in a way that is consistent with the mathematical and logical requirements as laid out by Cox [1] and Jaynes [2]. In particular, we have proved that the HSR is Maximum Entropy (**MaxEnt**), and thereby is also consistent with the Second Law of Thermodynamics.

For the simple case of only two equally probable hypotheses (or events) the conventional and hyperbolic versions of the sum rule yield similar (but not identical) results (see Figure 2). However, when large numbers of hypotheses are being considered, we have shown (Appendix A.5) that properties derived from the HSR are needed to obtain an appropriate Conventional Sum Rule (**CSR**). That is, although the CSR is applicable to certain simple cases, the HSR must be used where recursion cannot be ruled out (and where we need a MaxEnt solution in accordance with Bayesian requirements).

Apart from its mathematical consistency, we should also note empirically that numerous other physical phenomena involving probabilities also obey the HSR, as one might expect considering the (widely acknowledged) hyperbolic nature of the spacetime metric for the universe (see, for example, Penrose 2004 [24]). Therefore, we conclude that the Hyperbolic Sum Rule implies that *probability is physical* (also supported by the fact that the HSR is MaxEnt). This is because *probabilities* always refer to *phenomena* (however idealised). Phenomena belong in the real world, and their value (including measurement) is necessarily assessed by people for their own purposes. It is, therefore, satisfactory that the general MaxEnt case is represented by a formalism isomorphic to formalisms representing probabilities incorporated with, in general, physical systems (such as the etalon, Equation (1c)).

In terms of application, we expect that these methods will make analytically tractable those calculations that involve many hypotheses featuring multiple dependencies (both spatial and temporal). For example, disentangling the highly complex interactions of the different genes within a single genome represents an extremely difficult computational challenge, yet one with a glittering set of potential medicinal benefits. Another entirely different example regards the handling of certain important filters used in modern digital signal processing. Doubtless, others will also emerge.

Another application which exploits the handling of multiple hypotheses featuring highly interrelated, highly complex and recursive dependencies (currently thought either intractable or impracticable for explicit analytical evaluation) is in the emerging field of rule-based expert learning systems for inference. Such applications depend on the use of Bayesian reasoning, and exploit modern computational resources to handle a high degree of multi-modal inputs and outputs, each requiring a very high number of explicit (and implicit) variables (hypotheses) with associated conditional dependencies. However, issues of reliability, predictability and engineering control of AI technology are becoming more salient, since these are critical prerequisites for the systemic, safe, and validated use of AI technology. We are confident that the Hyperbolic Sum Rule will also contribute towards encouraging AI’s development as a scalable and high-valued technological asset.

## 6. Conclusions

In general, probabilities of events or hypotheses are added according to a “hyperbolic sum rule”. The linear rule that is conventional is applicable only in certain limited circumstances (where it is known that recursion is not present).

The new rule has been proved “Maximum Entropy”, that is, it is applicable to probability problems requiring unbiassed general solutions according to well-known Bayesian principles. It has been used to generalise the conventional rule for multiple hypotheses, to prove that it too is “Maximum Entropy”; albeit the CSR exhibits a systematically lower maximum entropy because of its extra assumptions. Various useful formulae have been derived for applicability to problems involving multiple hypotheses.

## Data Availability

The original contributions presented in this study are included in the article. Further inquiries can be directed to the corresponding author.

## Appendices: MaxEnt Properties of the Sum Rules for Probabilities

The Hyperbolic Sum Rule (**HSR**) for probabilities is intended to express the most likely (Maximum Entropy, **MaxEnt**) sum of probabilities where the conditional probabilities associated with the conventional sum rule are not available and where the recursive properties are also not explicitly known. That is to say, in the absence of the additional information (associated with the conditional probabilities as well as recursion) required to define the conventional sum rule, we can still define the most likely addition of the probabilities using MaxEnt methods. Note that, mathematically, there is no substantive difference between a function and the distribution it describes. We may regard either as “MaxEnt” when their entropy is maximised.

We investigate the MaxEnt properties of both the HSR and the Conventional Sum Rule (**CSR**). First (Appendix A.1) we establish a convenient criterion for proving that a probability function is MaxEnt, then (by way of example: Appendix A.2) we explore some simple functions. We prove (Appendix A.3) that the hyperbolic tangent sum rule is MaxEnt and, therefore, that the (isomorphic) HSR is also. In Appendix A.4 we derive sum rules for multiple hypotheses together with multiple conditionalities: these cases are complicated and require use of the HSR, being intractable on the assumptions of the CSR. In Appendix A.5 we show that the CSR is also MaxEnt for the limited cases where it applies, and show how the CSR should be concatenated to enable the handling of multiple hypotheses. We also show here that although both the HSR and the CSR are MaxEnt, the HSR has a higher entropy than the CSR, so that the HSR possesses fewer implicit constraints (that is, fewer implicit assumptions) than the CSR.

Finally, (Appendix A.6) we discuss how the HSR may find application in the engineering design of infinite impulse response (**IIR**) filters used in digital signal processing (**DSP**).

### Appendix A.1. Maximum Entropy Criterion for Sum Rules

Consider the function *Z* = *f*(*z*_0_, *z*_1_, *z*_2_ … *z_i_* … *z_N_*):

(A1a) $$Z=\sum_{i=0}^{N}z_{i}\equiv \sum_{i=0}^{N}e^{-\lambda i}$$

And hence the normalisation condition in which the terms are dimensionless:

(A1b) $$\sum_{i=0}^{N}\frac{z_{i}}{Z}=1$$

This is known to be MaxEnt (see Appendix I of Jaynes 1982 [3]): that is, the distribution it describes is demonstrably Maximum Entropy. It is also known to be equivalent to the Partition Function in statistical mechanics.

The free parameter *λ* is the essential controlling variable of the function *f*, expressing the ‘shape’ (or rate of exponential decay) of the function, while the index *i* is constrained to be an integer, with the maximum value *N* representing another free parameter of the function. The entropy S of the function *f* is given by a term-by-term summation of the Shannon entropy of each *i*th component:

(A2) $$S=-\sum_{i=0}^{N}\frac{z_{i}}{Z}\ln\frac{z_{i}}{Z}$$

Each *i*th component z*_i_* is associated with an *i*th state of the system *f*; that is to say, the function *f* can also be thought of as describing the relative ‘probability’ (or propensity for existence) of each of the components comprising a system of *N* different (distinguishable) states. In which case, the *i*th component *z_i_* can also be considered to represent a measure of the ‘relative strength’ of the *i*th component of the system. The fact that the system may be characterised with a finite entropy S means that we can assume that the number of states of the system is finite and has a granularity (that is, it is discretised, or quantised).

Consequently, this also means that there exists a quantised number *E*, representative of the overall system, where the discretisation (granularity) is represented by the quantum Δ*E*, such that the overall total number of quanta *Q* existing within the system is Q=E/ΔE. By inspection, it is also clear that Q≡Z. How these *Q* quanta are distributed across the function *f* represents a constraint to the system as we maximise its entropy. We therefore associate a quantity *E_i_* with each of the component terms of *f* such that the product of *z_i_* and *E_i_* for each component represents a measure of the strength of the *i*th state, and by summing across all the *N* + 1 states we obtain the overall quantity *E*:

(A3a) $$\sum_{i=0}^{N}z_{i}E_{i}=E$$

which can be re-written as:

(A3b) $$\sum_{i=0}^{N}\frac{z_{i}}{Z}E_{i}=\frac{E}{Z}=\frac{E}{Q}=\Delta E$$

Following the standard treatment of Caticha [4] we employ Lagrange multipliers *α* and λ, such that we set the independent equations to zero. We can then use Equations (A1b), (A2) and (A3b) to consider the variation in the system entropy S with *z_i_* so as to find its maximum:

(A4) $$\frac{\partial }{\partial z_{i}}\left\{\sum_{i=0}^{N}-\frac{z_{i}}{Z}\ln\frac{z_{i}}{Z}+\alpha \left(1-\sum_{i=0}^{N}\frac{z_{i}}{Z}\right)+\lambda \left(\Delta E-\sum_{i=0}^{N}\frac{z_{i}}{Z}E_{i}\right)\right\}=0$$

We can swap the order of the summation and differentiation operations to evaluate the derivative of each of the *i* terms in Equation (A4):

(A5) $$\sum_{i=0}^{N}\left(-\frac{1}{Z}\ln\frac{z_{i}}{Z}-\frac{1}{Z}-\frac{\alpha }{Z}-\frac{\lambda }{Z}E_{i}\right)=0$$

To maximise the system entropy S the *i*th component *z_i_* of the function *f* must be simply given by:

(A6) $$z_{i}=\frac{Z}{e^{1+\alpha }}e^{-\lambda E_{i}}$$

which is recognised as the canonical ensemble: a standard result, but obtained in a way that emphasises how “MaxEnt” it is.

Summarising, a distribution (or function) *f* is Maximum Entropy if its constituent terms follow a decaying exponential profile (see Equation (A1a)).

### Appendix A.2. Inadmissible “MaxEnt” Sum Rules

The most basic sum rule function *S*_0_(*A*,*B*) = *A* + *B* is Maximum Entropy (MaxEnt), since it is easy to demonstrate that the summation of any two terms can be made to follow the decaying exponential template of Equation (A6). For example:

(A7a) S0A,B=A+B=A1+BA≡Ze1+α1+e−λ

which is simply the two terms of a *N* = 1 series, for *i* = 0 and *i* = 1. That is to say, we can express the ratio *B*/*A* as being equivalent to exp(−*λ*) for an appropriate value of *λ*, while *A* is equivalent to *Z*/exp(1 + *α*) for an appropriate value of *α*. Of course, the converse is also true:

(A7b) S0A,B=A+B=B1+AB≡Ze1+α1+e−−λ

where for consistency, we employ the same value for *λ* as in Equation (A7a), but the values for *α* and *Z* are as appropriate.

The simplest sum rule of Equations (A7) is therefore easily found to be MaxEnt: are there any other sum rules that are also MaxEnt? Clearly, any such sum rule must conform to the template of Equation (A6) in order to be MaxEnt. The next obvious candidate that only employs the two parameters *A* and *B* is given by the following geometric progression of *N* + 1 terms:

(A8a) $$S_{1}\left(A,B\right)=A\left(1+\frac{B}{A}+{\left(\frac{B}{A}\right)}^{2}+{\left(\frac{B}{A}\right)}^{3}+\cdots +{\left(\frac{B}{A}\right)}^{N}\right)\equiv \frac{Z}{e^{1+\alpha }}\left(1+e^{-\lambda }+e^{-2\lambda }+e^{-3\lambda }+\cdots +e^{-N\lambda }\right)$$

There are two aspects to this sum rule that need to be noted: it only converges for *B* < *A* as *N*→∞, and it is clear that the overall summation can easily sum to greater than unity for various values of *A* and *B*. In particular, this latter aspect can be seen by re-writing Equation (A8a) as:

(A8b) $$S_{1}\left(A,B\right)=A\left(1+\frac{B}{A}+{\left(\frac{B}{A}\right)}^{2}+{\left(\frac{B}{A}\right)}^{3}+\cdots +{\left(\frac{B}{A}\right)}^{N}\right)=A+B+A{\left(\frac{B}{A}\right)}^{2}+A{\left(\frac{B}{A}\right)}^{3}+\cdots +A{\left(\frac{B}{A}\right)}^{N}$$

That is to say, the first two terms of the series come from the most basic sum rule, *S*_0_, and there then follow potentially an infinity of additional terms, such that the overall summation could well be greater than unity. Thus, as a general probability theoretical sum rule, it is inadmissible. Of course, the case where *A* < *B*, requires the converse sum rule:

(A8c) $$S_{2}\left(A,B\right)=B\left(1+\frac{A}{B}+{\left(\frac{A}{B}\right)}^{2}+{\left(\frac{A}{B}\right)}^{3}+\cdots +{\left(\frac{A}{B}\right)}^{N}\right)\equiv \frac{Z}{e^{1+\alpha }}\left(1+e^{-\lambda }+e^{-2\lambda }+e^{-3\lambda }+\cdots +e^{-N\lambda }\right)$$

These geometric progressions offer a closed form analytic solution:

(A9a) $$S_{1}\left(A,B\right)=A\frac{1-{\left(\frac{B}{A}\right)}^{N}}{1-\frac{B}{A}}=\frac{A^{2}}{A-B}\;\mathrm{as}\;N\to \infty \;\mathrm{for}\;A>B$$

and

(A9b) $$S_{2}\left(A,B\right)=B\frac{1-{\left(\frac{A}{B}\right)}^{N}}{1-\frac{A}{B}}=\frac{B^{2}}{B-A}\;\mathrm{as}\;N\to \infty \;\mathrm{for}\;B>A$$

Clearly, both forms for the sum rule have a pole (singularity) when *A* = *B*, such that the overall summation will be greater than unity. These are indeed ‘acceptable’ MaxEnt solutions as per Equation (A6), but are clearly deficient as *probability theoretical* sum rules, since the two rules each need to be judiciously selected (where disjoint application of the two rules is required according to which of *A* and *B* is the greater), and they can also sum to greater than one. We can therefore *exclude* all the rules *S*_0_, *S*_1_ and *S*_2_ as appropriate general MaxEnt probabilistic sum rules.

It is interesting to note that the conventional probability theoretical sum rule (employing conditional probabilities) always lies within the range of zero and one:

(A10) $$S_{CSR}\left(A,B\right)=A+B-A\times B_{|A}=A+B-B\times A_{|B}$$

That is to say, the first two terms of the Conventional Sum Rule (CSR) are equivalent to the basic sum rule function of Equations (A7), but there is an additional third term to the rule, which ensures that the overall quantity behaves ‘correctly’ as a probability quantity, such that it never exceeds unity. However, this is at the cost of introducing an additional term (indeed, additional information), alongside *A* and *B*: the conditional probabilities *B*_|*A*_ or *A*_|*B*_ (depending on which probability is considered prior). Such a conditional probability is essentially an additional (third) parameter to the sum rule, such that the function in effect becomes a sum rule requiring three input arguments, rather than just the two parameters (*A* and *B*) that we have been discussing above. From this perspective, the conventional probabilistic sum rule of Equation (A10) cannot be MaxEnt (apart from the fact that it does not follow the template of Equation (A6)), because it requires additional information (based on the conditional probabilities); whereas the rationale for a Maximum Entropy sum rule is to sum (in the most likely manner) the two parameters *A* and *B* in such a way that the minimal assumptions as to how *A* and *B* might depend upon or be related to each other is made; that is to say, the MaxEnt sum rule requires the least additional information over and above the values for *A* and *B* so as to provide their *most likely* sum value in the absence of any other information.

It is worth noting that when the events *A* and *B* are mutually exclusive, then the conventional probabilistic sum rule of Equation (A10) degenerates to the basic sum rule of Equations (A7). However, although the basic sum rule is MaxEnt (as we have previously discussed) the underlying reason for the degeneracy in this case of Equation (A10) means that we now *know* the exact (mutually exclusive) relationship between *A* and *B*. In general, the conventional sum rule of Equation (A10) is *not* MaxEnt since it requires the additional knowledge (or a best-guess estimate) of the (conditional) relationship between *A* and *B*; that is, it contains three explicit quantities of information for the ostensible addition of two of those quantities. But for the special case where there is the knowledge that *A* and *B* are mutually exclusive, then this causes the conventional sum rule to degenerate to the basic sum rule (with its MaxEnt properties), but which is still inadmissible as a general probability theoretical sum rule since it frequently leads to probabilities greater than one. Appendix A.5, however, shows that in the limit where the conditional probabilities are assumed identical to the independent probabilities (itself a MaxEnt assumption), then the CSR becomes MaxEnt in its domain of applicability. In addition, Appendix A.5 shows that for the case where the CSR admits multiple hypotheses (and where its mathematical structure is assumed to be approximately the arithmetic analogue to the HSR) then it is also found to be MaxEnt.

### Appendix A.3. Hyperbolic Sum Rule Is MaxEnt

We seek to prove that *S_HSR_*(A,B) is MaxEnt, where

(A11a) $$S_{HSR}\left(A,B\right)=\frac{A+B}{1+AB}$$

and where {*A*,*B*} are events (or hypotheses) and A ≡ *p*(*A*), B ≡ *p*(*B*); that is, *p*(*A*) is the probability for event *A* to occur (or hypothesis *A* to be true). Equation (A11a) here is essentially the same as Equation (12).

That is, we seek to prove that Equation (A11a) may be written in the form of *Z* = Σ*z_i_* (Equation (A1a)), where *z_i_* are given by Equation (A6):

$$Z=\sum_{i=0}^{N}z_{i}\equiv \sum_{i=0}^{N}e^{-\lambda i}$$

$$z_{i}=\frac{Z}{e^{1+\alpha }}e^{-\lambda E_{i}}$$

Equation (A11a) is isomorphic to the hyperbolic tangent double-angle identity (Equation (1a)):

(A11b) $$\tanh\left(a+b\right)=\frac{\tanh a+\tanh b}{1+\tanh a\tanh b}$$

Using tanhθ=sinhθ/coshθ as well as $\sinh\theta =½\left\{\exp\theta -\exp\left(-\theta \right)\right\}$ and $\cosh\theta =½\left\{\exp\theta +\exp\left(-\theta \right)\right\}$ it is straightforward to re-write Equation (A11b) as follows:

(A12) $$\tanh\left(a+b\right)=\frac{\exp\left(a+b\right)-\exp\left(-\left(a+b\right)\right)}{\exp\left(a+b\right)+\exp\left(-\left(a+b\right)\right)}=\frac{1-\exp\left(-2\left(a+b\right)\right)}{1+\exp\left(-2\left(a+b\right)\right)}=\frac{1-e^{-\lambda }}{1+e^{-\lambda }}$$

where in this case $\lambda =2\left(a+b\right)$. The identity Equation (A11b) is composed of the quotient of two functions, each a sum conforming to Equation (A1a) with *N* = 1, so that both are MaxEnt.

We prove that the resulting (quotient) function is also MaxEnt by binomially expanding the denominator function of Equation (A12) as an infinite series:

(A13) $$\tanh\left(a+b\right)=\left(1-e^{-\lambda }\right){\left(1+e^{-\lambda }\right)}^{-1}=\left(1-e^{-\lambda }\right)\left(1-e^{-\lambda }+e^{-2\lambda }-e^{-3\lambda }+e^{-4\lambda }-\cdots \right)$$

(A14) $$\;=1-2e^{-\lambda }+2e^{-2\lambda }-2e^{-3\lambda }+2e^{-4\lambda }-2e^{-5\lambda }+\cdots$$

(A15) $$\tanh\left(a+b\right)=-1+2\sum_{m=0}^{\infty }{\left(-e^{-\lambda }\right)}^{m}$$

(A16) $$\;=2\sum_{m=0}^{N}\left({\left(-e^{-\lambda }\right)}^{m}-\frac{1}{2N}\right)\;as\;N\to \infty$$

where

(A17) $$z_{m}=2{\left(-e^{-\lambda }\right)}^{m}-\frac{1}{N}\to z_{m}=2{\left(-e^{-\lambda }\right)}^{m}\;as\;N\to \infty$$

Therefore, tanh(*a* + *b*) is MaxEnt and the isomorphic HSR (Equation (A11a)) is also MaxEnt.

The HSR is a well-behaved probability theoretical quantity, since it always unconditionally yields results between zero and one for the sum of any conventional probability quantities (real values between zero and one inclusive).

Appendix A.5 additionally shows that the HSR always has a higher maximum entropy than the CSR, since the HSR involves fewer assumptions than the CSR.

### Appendix A.4. MaxEnt Treatment of HSR Featuring Multiple Hypotheses

#### Appendix A.4.1. ‘OR’ Treatment of Multiple Conditional Hypotheses

We wish to prove a type of Bayes Theorem (Equation (2c)) for multiple conditional hypotheses. Writing {*X*}_or_ for the sum of multiple hypotheses (*B* or *C* or *D* or …), then as the number of these hypotheses increases as follows:

(A18) $$p\left(A\right)p\left(\left.{\left\{X\right\}}_{\mathrm{OR}}\right|A\right)=p\left({\left\{X\right\}}_{\mathrm{OR}}\right)p\left(\left.A\right|{\left\{X\right\}}_{\mathrm{OR}}\right)\;\mathrm{as}\;\left\{X\right\}\to \infty$$

This result could be thought trivial, but it is obtained from a systematic approach to concatenating hypotheses which is an extension (Equation (A19)) of the trigonometry identity, Equation (1a) (Equation (A11b) in Appendix A.3) giving the isomorphic Equation (A20).

Since the Hyperbolic Sum Rule is isomorphic to the hyperbolic tangent double-angle identity it immediately suggests how additional hypotheses can be added, while continuing to ensure a logical and consistent treatment (exploiting the method discussed by Cox [1]), and maintaining its intrinsic MaxEnt characteristic (see Appendix A.3). In particular, considering an additional hypothesis *D* (with its own set of dependencies upon the hypotheses *A* and *B*) being added to the present scenario, the following identity result can now be exploited as follows:

(A19) $$\begin{array}{ll} \tanh\left\{\left(A+B\right)+D\right\} & \equiv \frac{\tanh\left(A+B\right)+\tanh\left(D\right)}{1+\tanh\left(A+B\right)\tanh\left(D\right)}\\ & =\frac{\tanh A+\tanh B+\tanh D+\tanh A\tanh B\tanh D}{1+\tanh A\tanh B+\tanh A\tanh D+\tanh B\tanh D} \end{array}$$

Since the addition (OR) function is associative, it is clear that all the terms in Equation (A19) must be mutually symmetrical, such that any other pair could have been chosen as the first argument, with an equivalent result. Therefore, we can write the probability for a set of three hypotheses *A*, *B* and *D* as follows:

(A20) $$p\left(\left.A\;OR\;B\;OR\;D\right|C\right)=\frac{p\left(\left.A\right|C\right)+p\left(\left.B\right|C\right)+p\left(\left.D\right|C\right)+p\left(\left.A\right|C\right)p\left(\left.B\right|C\right)p\left(\left.D\right|C\right)}{1+p\left(\left.A\right|C\right)p\left(\left.B\right|C\right)+p\left(\left.A\right|C\right)p\left(\left.D\right|C\right)+p\left(\left.B\right|C\right)p\left(\left.D\right|C\right)}$$

It should be noted that the conditionalities *C* associated with each probability are generic; but the probabilities employed in Equation (A20) for the explicit events *A*, *B*, and *D* are the *independent* probabilities for each of those events. That is to say, Equation (A20) is the MaxEnt expression for the HSR featuring three hypotheses, where no explicit knowledge about the dependencies between the three hypotheses is available.

Considering the RHS of Equation (A20), the first group of (single) terms in the numerator represent the independent probabilities {*p*(*A_n_*)}, while from a logical perspective, it is clear that the final (3rd-order) term of the numerator represents the product of these independent probabilities. Likewise, in the denominator, the three 2nd-order product terms (i.e., the 2nd-order permutations of the three hypotheses) each represent the symmetric probability products, as previously discussed. Clearly, if the third hypothesis is null, i.e., *p*(*D*) = 0, then from Bayes’ Theorem, all the terms containing *D* as a hypothesis also become zero, since *p*(*D*|*H*) = *p*(*D*) × *p*(*H*|*D*)/*p*(*H*), where *H* may represent any of the combinations of the hypotheses *A* and *B*, in which case, Equation (A20) reverts back to the two-hypotheses form (Equation (12)).

It is noteworthy that Equation (A19) also shows that multiple conditional hypotheses are to be treated using the OR function. For example, keeping the logical pair (*A* or *B*) distinct from *D*, and using the form of Equation (12), if we have explicit knowledge of the conditional probabilities, we can re-write Equation (A19) as follows:

(A21a) $$p\left(\left(A\;OR\;B\right)\;OR\;D\right)=\frac{p\left(A\;OR\;B\right)+p\left(D\right)}{1+p\left(\left.A\;OR\;B\right|D\right)p\left(\left.D\right|\left(A\;OR\;B\right)\right)}$$

Expanding out the paired (*A* or *B*) terms using the *tanh* identity of Equation (1a) as template, we can further write:

(A21b) $$p\left(\left(A\;OR\;B\right)\;OR\;D\right)=\frac{\frac{p\left(A\right)+p\left(B\right)}{1+p\left(\left.A\right|B\right)p\left(\left.B\right|A\right)}+p\left(D\right)}{1+\frac{p\left(\left.A\right|D\right)+p\left(\left.B\right|D\right)}{1+p\left(\left.A\right|\left(B\;OR\;D\right)\right)p\left(\left.B\right|\left(A\;OR\;D\right)\right)}p\left(\left.D\right|\left(A\;OR\;B\right)\right)}$$

In order to simplify the above equation, we need the conditionalities of the product terms to be identical whenever they appear in Equation (A21b); since all the respective terms are required by considerations of symmetry and maximum entropy to be interchangeable. Therefore, we re-write Equation (A21b), as follows:

(A21c) $$p\left(A\;OR\;B\;OR\;D\right)=\frac{\frac{p\left(A\right)+p\left(B\right)}{1+p\left(\left.A\right|B\;OR\;D\right)p\left(\left.B\right|A\;OR\;D\right)}+p\left(D\right)}{1+\frac{p\left(\left.A\right|D\right)+p\left(\left.B\right|D\right)}{1+p\left(\left.A\right|\left(B\;OR\;D\right)\right)p\left(\left.B\right|\left(A\;OR\;D\right)\right)}p\left(\left.D\right|\left(A\;OR\;B\right)\right)}$$

where the inclusion of the additional conditionalities is indicated in bold for clarity. These additions here are justified by requiring symmetry. Simplifying:

(A21d) $$p\left(A\;OR\;B\;OR\;D\right)=\frac{p\left(A\right)+p\left(B\right)+p\left(D\right)+p\left(D\right)p\left(\left.A\right|\left(B\;OR\;D\right)\right)\left(\left.B\right|\left(A\;OR\;D\right)\right)}{1+p\left(\left.A\right|\left(B\;OR\;D\right)\right)p\left(\left.B\right|\left(A\;OR\;D\right)\right)+\left[p\left(\left.A\right|D\right)+p\left(\left.B\right|D\right)\right]p\left(\left.D\right|\left(A\;OR\;B\right)\right)}$$

or, imposing the necessary symmetries:

(A21e) $$p\left(A\;OR\;B\;OR\;D\right)=\frac{p\left(A\right)+p\left(B\right)+p\left(D\right)+p\left(\left.D\right|(A\;OR\;B)\right)p\left(\left.A\right|\left(B\;OR\;D\right)\right)p\left(\left.B\right|\left(A\;OR\;D\right)\right)}{1+p\left(\left.A\right|\left(B\;OR\;D\right)\right)p\left(\left.B\right|\left(A\;OR\;D\right)\right)+\left[p\left(\left.A\right|\left(D\;OR\;B\right)\right)+p\left(\left.B\right|\left(A\;OR\;D\right)\right)\right]p\left(\left.D\right|\left(A\;OR\;B\right)\right)}$$

If the conditional probabilities are *not* known, then the appropriate MaxEnt version of Equation (A21e) (where no extraneous assumptions or inadvertent information is added) is required, whereby only the independent probabilities are employed as appropriate, and as seen in Equation (A20).

The probability of a hypothesis being true only requires at least one of the multiplicity of conditional hypotheses (say the set {*X*}_or_) to be also true. This means that the more the number of conditional hypotheses, say the set {*X*}, that a given hypothesis *A* is conditional upon, then the higher is the conditional probability that *A* will be true. That is: $p\left(\left.A\right|{\left\{X\right\}}_{\mathrm{OR}}\right)\to p\left(A\right)\;\mathrm{as}\;\left\{X\right\}\to \infty$.

The converse is also true: as the number of conditional hypotheses increases, they ‘crowd out’ the lone hypothesis *A*, and the probability of at least one of the conditional hypotheses {*X*} being true is increasingly independent of *A*: $p\left(\left.{\left\{X\right\}}_{\mathrm{OR}}\right|A\right)\to p\left({\left\{X\right\}}_{\mathrm{OR}}\right)\;\mathrm{as}\;\left\{X\right\}\to \infty$. Therefore:

(A22) $$\frac{p\left(\left.{\left\{X\right\}}_{\mathrm{OR}}\right|A\right)}{p\left({\left\{X\right\}}_{\mathrm{OR}}\right)}\to 1\;\mathrm{and}\;\frac{p\left(\left.A\right|{\left\{X\right\}}_{\mathrm{OR}}\right)}{p\left(A\right)}\to 1\;\mathrm{as}\;\left\{X\right\}\to \infty$$

Equating the two expressions offers us a type of Bayes’ Theorem for multiple contingent hypotheses, and we recover Equation (A18) as required:

$$p\left(A\right)p\left(\left.{\left\{X\right\}}_{\mathrm{OR}}\right|A\right)=p\left({\left\{X\right\}}_{\mathrm{OR}}\right)p\left(\left.A\right|{\left\{X\right\}}_{\mathrm{OR}}\right)\;\mathrm{as}\;\left\{X\right\}\to \infty$$

Unfortunately, the same is not necessarily true for a low number of hypotheses {*X*}; however, for only two hypotheses, *A* and *X*, we recover Bayes’ Theorem (Equation (2c)).

#### Appendix A.4.2. Generalisation of the Concatenated “HSR” for Multiple Hypotheses

For a more convenient overview of the emerging ‘pattern’ as more hypotheses are concatenated, we may re-write Equation (A20) using only the logical hypotheses (and making the conditional hypotheses {*C*} implicit) thus:

$$p\left(A\;OR\;B\;OR\;D\right)=\frac{A+B+D+ABD}{1+AB+AD+BD}$$

where A ≡ *p*(*A*) etc. as before. Adding a fourth hypothesis, *E*, the hyperbolic tangent identity becomes the following:

(A23a) $$\tanh\left\{\left(A+B+D\right)+E\right\}\equiv \frac{\tanh\left(A+B+D\right)+\tanh\left(E\right)}{1+\tanh\left(A+B+D\right)\tanh\left(E\right)}$$

Again, only writing the symbols of the hypotheses for convenience, we can derive the associated hyperbolic sum rule for *four* such hypotheses as follows:

(A23b) $$p\left(A\;OR\;B\;OR\;D\;OR\;E\right)=\frac{\frac{A+B+D+ABD}{1+AB+AD+BD}+E}{1+\frac{A+B+D+ABD}{1+AB+AD+BD}E}=\frac{A+B+D+E+ABD+ABE+ADE+BDE}{1+AB+AD+BD+AE+BE+DE+ABDE}$$

For clarity, we remind ourselves that the final term of the denominator of Equation (A23b) is to be understood as follows:

$$ABDE\equiv p\left(\left.A\right|\left(B\;OR\;D\;OR\;E\right)\right)p\left(\left.B\right|\left(A\;OR\;D\;OR\;E\right)\right)p\left(\left.D\right|\left(A\;OR\;B\;OR\;E\right)\right)p\left(\left.E\right|\left(A\;OR\;B\;OR\;D\right)\right),$$

where we assume here that the conditional probabilities are known. If, however, the conditional probabilities are not known, then the independent probabilities need to be employed as per the MaxEnt requirements of a Bayesian analysis.

#### Appendix A.4.3. Sum Rule for Multiple Hypotheses with Multiple Conditionalities

We can also use the hyperbolic tangent function to analyse the issue of calculating the MaxEnt probability of a set of multiple hypotheses that are conditionally dependent on an additional set of hypotheses. For example, the simplest such ‘multiple’ scenario is given by the probability quantity $p\left(\left.A\;OR\;B\right|D\;OR\;E\right)$, which represents the probability of hypotheses *A* or *B* being correct, given that they are conditionally dependent on the hypotheses *D* or *E*. Using Equation (12) and the probability calculus of the section Appendix A.4.1, this can be expressed as follows:

(A24) $$p\left(\left.A\;OR\;B\right|D\;OR\;E\right)=\frac{p\left(\left.A\right|D\;OR\;E\right)+p\left(\left.B\right|D\;OR\;E\right)}{1+p\left(\left.A\right|B\;OR\;D\;OR\;E\right)p\left(\left.B\right|A\;OR\;D\;OR\;E\right)}$$

Clearly, the complexity of the overall expression increases rapidly with increasing number of hypotheses and conditional dependencies, with the attendant multitude of dependency terms and cross-products. However, we have shown here that such a calculation is indeed analytically possible, and the MaxEnt method for analysing higher numbers of hypotheses with their conditionalities is clearly apparent. This simple and generalisable result is not available from the conventional sum rule.

#### Appendix A.4.4. Generalised Hyperbolic Sum Rule for Multiple Hypotheses

The hyperbolic sum rule sorts out all the odd and even combinations of the hypotheses, such that the odd-ordered conjunction products are located in the numerator, while the even-ordered products are in the denominator. This can be seen in Equation (A20) for three hypotheses: *p*(*A* or *B* or *C*); and Equation (A23b) for four hypotheses: *p*(*A* or *B* or *C* or *D*).

Each of the elements in the numerator and denominator corresponds to an element of the binomial expansion of the expression (*a* + *b*)*^N^*, where *N* represents the number of hypotheses; there being a total of 2*^N^* different and unique combinations of the various hypotheses, and there being $C_{n}^{N}\equiv N!/n!\left(N-n\right)!$ combinatorial elements associated with each order of conjunction.

Then, the general expression for the Hyperbolic Sum Rule involving *N* different hypotheses each with their various mutual Bayesian dependencies is given by the following:

(A25) $$p\left(A_{1}\;OR\;A_{2}\;OR\;\ldots \;OR\;A_{N}\right)=\frac{\sum_{l=1,\mathrm{odd}}^{N}\sum_{m=1}^{m=\frac{N!}{l!\left(N-l\right)!}}\prod_{n=P\left\{l,1:N\right\}}^{l}p\left(\left.A_{n}\right|\left\{A_{n^{\prime }},l-1\right\}\right)}{\sum_{l=0,\mathrm{even}}^{N}\sum_{m=1}^{m=\frac{N!}{l!\left(N-l\right)!}}\prod_{n=P\left\{l,1:N\right\}}^{l}p\left(\left.A_{n}\right|\left\{A_{n^{\prime }},l-1\right\}\right)}$$

where the conditionality part of the probability $p\left(\left.A_{n}\right|\left\{A_{n^{\prime }},l-1\right\}\right)$ is given by the term in the curly brackets $\left\{A_{n^{\prime }},l-1\right\}$ of Equation (A25) which indicates that there are *l* − 1 conditional hypotheses associated with the hypothesis *A_n_*; these conditional hypotheses to be selected from the set $\left\{A_{n^{\prime }}\right\}$, where the prime in the subscript indicates that the hypothesis *A_n_* is itself excluded from the set. These conditional hypotheses (which, of course, exclude the hypothesis under question *A_n_*) must be selected in the appropriate permutations from the set of *N* − 1 probabilities making up the rest of the set $\left\{p\left(A_{n^{\prime }}\right)\right\}$. It is also clear that as the number of conditional hypotheses *l* − 1 becomes large, then it is also reasonable to start making the simplifying approximation $p\left(\left.A_{n}\right|\left\{A_{n^{\prime }},l-1\right\}\right)\approx p\left(A_{n}\right)$ as *l*→∞ becomes valid (see Appendix A.4.1 for multiple hypotheses).

We note that the case for *l* = 0 (the first even term) in the denominator of Equation (A25) is simply the constant term of unity (‘1’) always found in the denominator of the HSR equation, as already seen in Equations (A21e) and (A24). In addition, the function *P*{*l*,1:*N*} associated with the product (Π) terms in Equation (A25) represents the selection of the complete set of all possible (and distinct) permutated product terms (of overall degree *l*), each consisting of the product of *l* distinct probability terms from the set of *N* probabilities {*p*(*A_n_*)}.

We prove further that as the number of hypotheses increases without limit, at Maximum Entropy, the formula of Equation (A25) is well-behaved. At MaxEnt where each hypothesis (and each conditional dependency) is equally possible, *p*(*A_n_*) = 1/*N*, and *p*(*A_n_*|{*A_n’_*}) = 1/*N*. Then:

(A26) $$p\left(A_{1}\;OR\;A_{2}\;OR\;\ldots \;OR\;A_{N}\right)=\frac{\sum_{l=1,\mathrm{odd}}^{N}\frac{N!}{l!\left(N-l\right)!}\prod_{n=1}^{l}p\left(A_{n}\right)}{\sum_{l=0,\mathrm{even}}^{N}\frac{N!}{l!\left(N-l\right)!}\prod_{n=1}^{l}p\left(A_{n}\right)}=\frac{\sum_{l=1,\mathrm{odd}}^{N}\frac{N!}{l!\left(N-l\right)!}\frac{1}{N^{l}}}{\sum_{l=0,\mathrm{even}}^{N}\frac{N!}{l!\left(N-l\right)!}\frac{1}{N^{l}}}$$

We make use of the following binomial expansion identity:

(A27a) $${\left(1+\frac{1}{N}\right)}^{N}=\sum_{l=0}^{N}\frac{N!}{l!\left(N-l\right)!}\frac{1}{N^{l}}$$

In which case, the summations appearing in Equation (A26), respectively, consisting of the odd and even terms only, therefore each represent approximately half the amount on the LHS of Equation (A27a); this approximation being more accurate as *N* becomes large. Therefore:

(A27b) $$\sum_{l=1,odd}^{N}\frac{N!}{l!\left(N-l\right)!}\frac{1}{N^{l}}\approx \sum_{l=0,even}^{N}\frac{N!}{l!\left(N-l\right)!}\frac{1}{N^{l}}\approx \frac{1}{2}{\left(1+\frac{1}{N}\right)}^{N}\;\mathrm{for}\;N\gg 1$$

So that substituting Equation (A27b) into Equation (A26) we find the following:

(A27c) $$p\left(A_{1}\;OR\;A_{2}\;OR\;\ldots \;OR\;A_{N}\right)=\frac{\frac{1}{2}{\left(1+\frac{1}{N}\right)}^{N}}{\frac{1}{2}{\left(1+\frac{1}{N}\right)}^{N}}=1\;\mathrm{for}\;N\to \infty$$

It is also worth noting the identity ${\left(1+1/N\right)}^{N}\to e\;\mathrm{as}\;N\to \infty$, so that for large *N* we can also re-write Equation (A27c) as follows:

(A27d) $$p\left(A_{1}\;OR\;A_{2}\;OR\;\ldots \;OR\;A_{N}\right)=\frac{0.5e}{0.5e}=1\;\mathrm{as}\;N\to \infty$$

#### Appendix A.4.5. Maximum Entropy Analysis for “HSR” of Multiple Hypotheses

We have shown that the Hyperbolic Sum Rule (HSR) is well-behaved for multiple hypotheses as N→∞, assuming a uniform (equal) MaxEnt distribution for the probabilities of the set of *N* possible hypotheses. However, we can also undertake another MaxEnt analysis, but for a finite temperature-based distribution of probabilities. In particular, we assume that we can arrange the probabilities of the hypotheses as a set of probabilities in a descending order of probability that conforms to a negative exponential distribution; this is the classic MaxEnt distribution of probabilities (see Jaynes 1982 [3] and Equations (A1a) and (A6) here) that conforms to a negative exponential distribution.

Thus, we assume that the independent probability of the *n*th hypothesis *p*(*A_n_*) is given by the following:

(A28a) $$p\left(A_{n}\right)=\left\{e^{\beta }\left(\frac{1-e^{-\beta }}{1-e^{-N\beta }}\right)\right\}e^{-\beta n}$$

where *β* acts as the appropriate Lagrange multiplier for the MaxEnt distribution (and *β* = 0 in the previous section Appendix A.4). In passing, we note that Equation (A28a) conforms implicitly to the following:

(A28b) $$\sum_{n=1}^{N}e^{-\beta n}=e^{-\beta }\left(\frac{1-e^{-N\beta }}{1-e^{-\beta }}\right)\mathrm{such}\;\mathrm{that}\sum_{n=1}^{N}p\left(A_{n}\right)=1$$

Substituting Equation (A28a) into Equation (A27a), we obtain the following:

(A28c) $$p\left(A_{1}\;OR\;A_{2}\;OR\;\ldots \;OR\;A_{N}\right)=\frac{\sum_{l=1,\mathrm{odd}}^{N}\frac{N!}{l!\left(N-l\right)!}{\left\{\left(\frac{1-e^{-\beta }}{1-e^{-N\beta }}\right)e^{\beta }\right\}}^{l}\prod_{n=P\left\{l,1:N\right\}}^{l}e^{-\beta n}}{\sum_{l=0,\mathrm{even}}^{N}\frac{N!}{l!\left(N-l\right)!}\frac{1}{N^{l}}{\left\{\left(\frac{1-e^{-\beta }}{1-e^{-N\beta }}\right)e^{\beta }\right\}}^{l}\prod_{n=P\left\{l,1:N\right\}}^{l}e^{-\beta n}}$$

which can be re-written:

(A28d) $$p\left(A_{1}\;OR\;A_{2}\;OR\;\ldots \;OR\;A_{N}\right)=\frac{\sum_{l=1,\mathrm{odd}}^{N}\frac{N!}{l!\left(N-l\right)!}{\left\{\left(\frac{1-e^{-\beta }}{1-e^{-N\beta }}\right)e^{\beta }\right\}}^{l}exp\left\{-\beta \sum_{n=P\left\{l,1:N\right\}}^{l}n\right\}}{\sum_{l=0,\mathrm{even}}^{N}\frac{N!}{l!\left(N-l\right)!}{\left\{\left(\frac{1-e^{-\beta }}{1-e^{-N\beta }}\right)e^{\beta }\right\}}^{l}exp\left\{-\beta \sum_{n=P\left\{l,1:N\right\}}^{l}n\right\}}$$

Note that the final product term (now transformed into a summation) at the end of both the numerator and denominator, corresponds to the permutated choices of all the *l* probability terms being considered for the *l*th-order conjunction. We can make a MaxEnt (Bayesian) approximation for this by assuming that the average value of *n* for any given choice is simply *N*/2. In which case, choosing *l* such instances means that the average (expected) summation is then simply $\bar{n}=lN/2$. Thus, we can approximate Equation (A28d) as follows:

(A28e) $$p\left(A_{1}\;OR\;A_{2}\;OR\;\ldots \;OR\;A_{N}\right)\approx \frac{\sum_{l=1,\mathrm{odd}}^{N}\frac{N!}{l!\left(N-l\right)!}{\left\{\left(\frac{1-e^{-\beta }}{1-e^{-N\beta }}\right)e^{\beta }\right\}}^{l}e^{-\frac{\beta lN}{2}}}{\sum_{l=0,\mathrm{even}}^{N}\frac{N!}{l!\left(N-l\right)!}{\left\{\left(\frac{1-e^{-\beta }}{1-e^{-N\beta }}\right)e^{\beta }\right\}}^{l}e^{-\frac{\beta lN}{2}}}$$

Simplifying:

(A28f) $$p\left(A_{1}\;OR\;A_{2}\;OR\;\ldots \;OR\;A_{N}\right)\approx \frac{\sum_{l=1,\mathrm{odd}}^{N}\frac{N!}{l!\left(N-l\right)!}{\left\{\left(\frac{1-e^{-\beta }}{1-e^{-N\beta }}\right)e^{-\beta \left(\frac{N}{2}-1\right)}\right\}}^{l}}{\sum_{l=0,\mathrm{even}}^{N}\frac{N!}{l!\left(N-l\right)!}{\left\{\left(\frac{1-e^{-\beta }}{1-e^{-N\beta }}\right)e^{-\beta \left(\frac{N}{2}-1\right)}\right\}}^{l}}$$

Using the approximation result of Equation (A27c) for large *N*, we can approximate the numerator as:

(A28g) $$\begin{array}{c} \sum_{l=1,odd}^{N}\frac{N!}{l!\left(N-l\right)!}{\left\{\left(\frac{1-e^{-\beta }}{1-e^{-N\beta }}\right)e^{-\beta \left(\frac{N}{2}-1\right)}\right\}}^{l}\approx \frac{1}{2}{\left\{1+\left(\frac{1-e^{-\beta }}{1-e^{-N\beta }}\right)e^{-\beta \left(\frac{N}{2}-1\right)}\right\}}^{N}\\ =\frac{1}{2}{\left\{1+\frac{e^{-\frac{\beta }{2}}}{e^{-\frac{N\beta }{2}}}\left(\frac{e^{\frac{\beta }{2}}-e^{-\frac{\beta }{2}}}{e^{\frac{N\beta }{2}}-e^{-\frac{N\beta }{2}}}\right)e^{-\beta \left(\frac{N}{2}-1\right)}\right\}}^{N}=\frac{1}{2}{\left\{1+\left(\frac{\sinh\beta /2}{\sinh N\beta /2}\right)e^{\frac{\beta }{2}}\right\}}^{N} \end{array}$$

Clearly for β=0, we can assume that $\sinh\left(\beta /2\right)/\sinh\left(N\beta /2\right)=1/N$ (see Equations (A26) and (A27)), so we revert to the initial uniform distribution of Equation (A27b). However, for β≠0 and N→∞, but β≪N, then we can approximate Equation (A28g) to:

(A28h) $$\frac{1}{2}{\left\{1+\left(\frac{\sinh\beta /2}{\sinh N\beta /2}\right)e^{\beta /2}\right\}}^{N}\approx \frac{1}{2}{\left\{1+\frac{e^{\beta /2}}{N}\right\}}^{N}\approx \frac{1}{2}\exp\left\{e^{\beta /2}\right\}$$

We can assume the same for the even-ordered series of the denominator, such that substituting into Equation (A27a) we have:

(A28i) $$p\left(A_{1}\;OR\;A_{2}\;OR\;\ldots \;OR\;A_{N}\right)\approx \frac{0.5\exp\left\{e^{\beta /2}\right\}}{0.5\exp\left\{e^{\beta /2}\right\}}=1\;\mathrm{as}\;N\to \infty$$

Equation (A28i) represents the MaxEnt generalisation for the Hyperbolic Sum Rule (HSR) as *N* gets very large. It can be seen that the HSR still remains well behaved as the Lagrange multiplier (inverse-temperature parameter) *β* is varied for the distributions of the set {*p*(*A_n_*)}, with *β* representing distributions associated with different effective temperatures. We have already shown an example of this in resolving the Wine-Water Paradox [38].

### Appendix A.5. Generalising the Conventional Sum Rule (CSR)

Using results obtained from deriving the HSR, we obtain a general CSR relation for *N* hypotheses (Appendix A.5.1, Equation (A34) below). We then confirm that the CSR is MaxEnt (Appendix A.5.2), and show that the CSR entropy is never greater than the HSR entropy (Appendix A.5.3). This latter is expected because the CSR has more constraints than does the HSR.

#### Appendix A.5.1. Concatenation Rules for CSR

We explore here how the *conventional sum rule* (CSR) behaves as the number of hypotheses considered increases. In particular, Equation (2b) (together with the HSR formalism of Equation (A25)) indicates a possible (but actually, naïve and erroneous, as we shall see) concatenation rule for the CSR featuring multiple hypotheses:

(A29) $$p\left(A_{1}\;OR\;A_{2}\;OR\;\ldots \;OR\;A_{N}\right)=\sum_{n=1}^{N}p\left(A_{n}\right)-\sum_{l=2}^{N}\sum_{m=1}^{m=\frac{N!}{l!\left(N-l\right)!}}\prod_{n=P\left\{l,1:N\right\}}^{l}p\left(\left.A_{n}\right|\left\{A_{n^{\prime }},l-1\right\}\right)$$

where the final double-summation and product term is recognised to consist of all the possible combinations (at all orders) for two or more conjuncting terms (that is, for l≥2).

However, for the case when each hypothesis is equally possible and each conditional dependency is equal to the independent probability (*p*(*A_n_*|{*A_n′_*}) = 1/*N*; and *p*(*A_n_*) = 1/*N*) then:

(A30) $$p\left(A_{1}\;OR\;A_{2}\;OR\;\ldots \;OR\;A_{N}\right)=\sum_{n=1}^{N}\frac{1}{N}-\sum_{l=2}^{N}\frac{N!}{l!\left(N-l\right)!}\frac{1}{N^{l}}$$

The second summation of Equation (A30) (which only starts at *l* = 2) may be rewritten as follows:

(A31) $$\sum_{l=2}^{N}\frac{N!}{l!\left(N-l\right)!}\frac{1}{N^{l}}\equiv \sum_{l=0}^{N}\frac{N!}{l!\left(N-l\right)!}\frac{1}{N^{l}}-\sum_{n=1}^{N}\frac{1}{N}-1=\sum_{l=0}^{N}\frac{N!}{l!\left(N-l\right)!}\frac{1}{N^{l}}-2$$

and therefore:

(A32) $$\begin{array}{c} p\left(A_{1}\;OR\;A_{2}\;OR\;\ldots \;OR\;A_{N}\right)=1-\sum_{l=2}^{N}\frac{N!}{l!\left(N-l\right)!}\frac{1}{N^{l}}=1-\left(\sum_{l=0}^{N}\frac{N!}{l!\left(N-l\right)!}\frac{1}{N^{l}}-2\right)\\ =3-\sum_{l=0}^{N}\frac{N!}{l!\left(N-l\right)!}\frac{1}{N^{l}}=3-{\left(1+\frac{1}{N}\right)}^{N}=3-e\;\mathrm{as}\;N\to \infty \end{array}$$

Since Equation (A32) tends to a non-unity result (3 − *e* = 0.282) as *N* becomes large Equation (A32) is not well behaved as a reliable probability quantity (and consequently Equation (A29) is also unsatisfactory). In which case, it is clear that the ‘possible’ CSR concatenation rule of Equation (A29) is inadmissible.

However, in the hyperbolic sum rule case (Equation (A25)) the odd- and even-ordered conjunctions are treated differently, with the even terms all sorted into the denominator and the odd terms located in the numerator. (This is the reason why Jaynes suggested that the Venn diagram of Figure 1 can ‘mislead’ since such respective groupings of the odd- and even-ordered overlaps is not obvious.) Applying this alternative (HSR-inspired) model to Equation (A29), and then subsequently to Equation (A32), such that the even-ordered terms are negative and the odd-ordered terms add positively, we have the following:

(A33) $$\begin{array}{ll} p\left(A_{1}\;OR\;A_{2}\;OR\;\ldots \;OR\;A_{N}\right) & =1-\sum_{l=2,even}^{N}\frac{N!}{l!\left(N-l\right)!}\frac{1}{N^{l}}+\sum_{l=3,odd}^{N}\frac{N!}{l!\left(N-l\right)!}\frac{1}{N^{l}}\\ & =\sum_{l=1,odd}^{N}\frac{N!}{l!\left(N-l\right)!}\frac{1}{N^{l}}-\sum_{l=2,even}^{N}\frac{N!}{l!\left(N-l\right)!}\frac{1}{N^{l}}\\ & =\frac{1}{2}{\left(1+\frac{1}{N}\right)}^{N}-\left[\frac{1}{2}{\left(1+\frac{1}{N}\right)}^{N}-1\right]\\ & =1-0.5e+0.5e=1 \end{array}$$

Here, we recognise that the first term of the CSR (looking at the RHS of the first line of Equation (A33)) is equivalent to the *l* = 1 term of the odd series of the first line: this term can be transferred to the odd series, extending its range. We also recognise that the even series is defined from *l* = 2, that is to say, it omits the *l* = 0 component.

However, in using the approximations of Equation (A27c) to calculate a reasonable closed-form for the series, we use the even series summation, and take away 1 in order to appropriately observe the omission of the *l* = 0 component. Note the subtle ambiguity for why ‘1’ in the first line of Equation (A33) (on the RHS) is present: is it due to the *l* = 1 term of the odd series or is it an artefact of the *l* = 0 term of the even series (as seen in the final line of Equation (A25))?

In any case, the CSR is now well behaved as the number of hypotheses increases. It seems that the general form of the CSR should be given by the following:

(A34) $$\begin{array}{c} p\left(A_{1}\;OR\;A_{2}\;OR\;\ldots \;OR\;A_{N}\right)=\sum_{l=1,odd}^{N}\sum_{m=1}^{m=\frac{N!}{l!\left(N-l\right)!}}\prod_{n=P\left\{l,1:N\right\}}^{l}p\left(\left.A_{n}\right|\left\{A_{n^{\prime }},l-1\right\}\right)-\sum_{l=2,even}^{N}\sum_{m=1}^{m=\frac{N!}{l!\left(N-l\right)!}}\prod_{n=P\left\{l,1:N\right\}}^{l}p\left(\left.A_{n}\right|\left\{A_{n^{\prime }},l-1\right\}\right)\\ =1+\sum_{l=1,odd}^{N}\sum_{m=1}^{m=\frac{N!}{l!\left(N-l\right)!}}\prod_{n=P\left\{l,1:N\right\}}^{l}p\left(\left.A_{n}\right|\left\{A_{n^{\prime }},l-1\right\}\right)-\sum_{l=0,even}^{N}\sum_{m=1}^{m=\frac{N!}{l!\left(N-l\right)!}}\prod_{n=P\left\{l,1:N\right\}}^{l}p\left(\left.A_{n}\right|\left\{A_{n^{\prime }},l-1\right\}\right) \end{array}$$

But it is fascinating that, in order to make the CSR behave reasonably for multiple hypotheses, key results have had to be borrowed from the HSR (i.e., that the odd and even product (conjunction) terms take different signs). The top line of Equation (A34) can therefore be considered to represent the *arithmetic* version of the HSR (Equation (A25), apart from the fact that the even summation only runs from *l* ≥ 2). Thus, the {CSR, HSR} can be approximately considered as {arithmetic, geometric} analogues.

The HSR is consistent with many other physical phenomena, and it has also allowed us to find Equation (A34). The HSR of Equation (A25) elegantly employs *all* the terms of the complete series summation without exception, whereas the CSR has to omit the first *l* = 0 term from the even series (unless the additional ‘unity’ term is introduced) as seen in the last line of Equation (A34). In addition, the hyperbolic sum rule does not require a change in sign of the conjunction terms, since they are all positive as they appear in Equation (A25); this is in contrast to Equation (A34) where a change in sign for the odd and even conjunction terms is necessary. Without the insights of the hyperbolic nature of Equation (A25), and, in particular, the properties of the multi-angle hyperbolic tangent function, it would not be possible to derive the form of Equation (A34) so easily. Note also that the generalised CSR for multiple hypotheses (Equation (A34)) still offers results relatively close to the HSR, e.g., as seen in Figure 2.

#### Appendix A.5.2. CSR Is MaxEnt in Its (Non-Recursive) Domain of Application

Finally, a discussion on whether the CSR of Equation (A34) is a MaxEnt function, both in general, and also when the conditional probabilities are assumed equal to the independent probabilities (this assumption also conforming to MaxEnt criteria). To decide this, we exploit the fact that the CSR (Equation (A34)) is an arithmetic version of the (already proven MaxEnt) HSR (Equation (A25)). That is to say, we know that the HSR of Equation (A25) is a quotient quantity:

(A35) $$p{\left(A_{1}\;OR\;A_{2}\;OR\;\ldots \;OR\;A_{N}\right)}_{HSR}=\frac{\sum_{l=1,\mathrm{odd}}^{N}\sum_{m=1}^{m=\frac{N!}{l!\left(N-l\right)!}}\prod_{n=P\left\{l,1:N\right\}}^{l}p\left(\left.A_{n}\right|\left\{A_{n^{\prime }},l-1\right\}\right)}{\sum_{l=0,\mathrm{even}}^{N}\sum_{m=1}^{m=\frac{N!}{l!\left(N-l\right)!}}\prod_{n=P\left\{l,1:N\right\}}^{l}p\left(\left.A_{n}\right|\left\{A_{n^{\prime }},l-1\right\}\right)}\equiv \frac{K}{L}$$

where we define the quantity *K* to represent the summation of the odd conjunctions, while *L* represents the summation of the even conjunctions. In which case, from Equation (A34) it is clear that we can simply represent the CSR as:

(A36) $$p{\left(A_{1}\;OR\;A_{2}\;OR\;\ldots \;OR\;A_{N}\right)}_{CSR}\equiv 1+K-L$$

In the previous Appendix A.4, e.g., Equation (A23), we can see that even when composed of multiple hypotheses the HSR can still be represented by a *single* hyperbolic tangent function, whose argument represents all the hypotheses to be summed. If we denote that summed argument as *γ*, then we can write:

(A37) $$p{\left(A_{1}\;OR\;A_{2}\;OR\;\ldots \;OR\;A_{N}\right)}_{HSR}\equiv \tanh\gamma$$

However, the hyperbolic tangent function *tanh* is itself an intrinsically quotient quantity, tanh(*γ*) ≡ sinh(*γ*)/cosh(*γ*), such that it is equally clear after comparing Equation (A35) with Equation (A37) that we can write:

*K* ≡ sinh(*γ*)    and    *L* ≡ cosh(*γ*) (A38)

We already demonstrated in Appendix A.3 that the HSR as represented by the hyperbolic tangent function is MaxEnt, but now with CSR ≡ 1 + *K* − *L* (according to Equation (A36)) then we can simply write for the multiple-hypotheses CSR:

(A39) $$p{\left(A_{1}\;OR\;A_{2}\;OR\;\ldots \;OR\;A_{N}\right)}_{CSR}\equiv 1+\sinh\gamma -\cosh\gamma \equiv 1+\frac{1}{2}\left(e^{\gamma }-e^{-\gamma }\right)-\frac{1}{2}\left(e^{\gamma }+e^{-\gamma }\right)=1-e^{-\gamma }$$

That is to say, CSR ≡ 1 − *e*^−*γ*^, which conforms to the MaxEnt template of Equation (A1a) discussed in Appendix A.1. Thus, it is clear that the Equation (A34) for the CSR is therefore also MaxEnt, albeit within its narrower domain of applicability (i.e., recursion is excluded).

#### Appendix A.5.3. HSR and CSR Entropies Compared

The entropies of the HSR and CSR can be readily compared. Considering the entropy S ≡ −*ρ*ln*ρ* − (1 − *ρ*)ln(1 − *ρ*) associated with the HSR, using Equation (A35) with the probability *ρ* given by *K*/*L* (and noting that the [1 − *ρ*] terms are needed in the expression for S since probabilities must sum to 1):

(A40) $$S_{HSR}=-\left(\frac{K}{L}\right)\ln\left(\frac{K}{L}\right)-\left(1-\frac{K}{L}\right)\ln\left(1-\frac{K}{L}\right)$$

Given that the quantities *K* and *L* represent, in general, the odd- and even-conjunction probability terms of a weighted binomial expansion, their sum always equal 1. In which case, as the terms *N* in the expansion get large, we can assume from Equation (A35) that for any such general set of multiple-hypotheses probabilities:

*K* = ½ − δ and *L* = ½ + δ (A41)

where δ ≪ 1 and is also a positive quantity, since it is clear we must have *K* ≤ *L* to ensure that Equation (A35) always represents a valid probability. In which case, the entropy of the HSR can be calculated using:

(A42a) $$-\left(\frac{K}{L}\right)\ln\left(\frac{K}{L}\right)=-\frac{½-\delta }{½+\delta }\ln\frac{½-\delta }{½+\delta }=-\frac{{\left(½-\delta \right)}^{2}}{¼-\delta^{2}}\ln\frac{{\left(½-\delta \right)}^{2}}{¼-\delta^{2}}\approx -\left(1-4\delta \right)\ln\left(1-4\delta \right)\approx \left(1-4\delta \right)4\delta$$

where we neglect the higher orders in δ, and also use the approximation for the natural logarithm function for small δ, ln(1 + δ) ≈ δ as δ→0. We also need:

(A42b) $$-\left(1-\frac{K}{L}\right)\ln\left(1-\frac{K}{L}\right)=-\left(\frac{L-K}{L}\right)\ln\left(\frac{L-K}{L}\right)=-\frac{2\delta }{½+\delta }\ln\frac{2\delta }{½+\delta }\approx -4\delta \ln4\delta$$

where we assume δ ≪ 1. This means the entropy of the HSR is:

(A42c) $$S_{HSR}\approx \left(1-4\delta \right)4\delta -4\delta \ln4\delta$$

The entropy of the CSR can be calculated using Equation (A36):

(A43) $$S_{CSR}=-\left(1+K-L\right)\ln\left(1+K-L\right)-\left(L-K\right)\ln\left(L-K\right)$$

Deploying the same values for *K* and *L* as given in Equation (A41), we see that the entropy of the CSR is calculated using:

(A44a) $$-\left(1+½-\delta -\left(½+\delta \right)\right)\ln\left(1+½-\delta -\left(½+\delta \right)\right)=-\left(1-2\delta \right)\ln\left(1-2\delta \right)\approx \left(1-2\delta \right)2\delta$$

and

(A44b) $$-\left(L-K\right)\ln\left(L-K\right)=-2\delta \ln2\delta$$

The entropy of the CSR is therefore closely given by:

(A44c) $$S_{CSR}\approx \left(1-2\delta \right)2\delta -2\delta \ln2\delta$$

Comparing term-by-term the two terms composing each entropy quantity, it is clear that for δ ≪ 1 (which also conforms with δ < 1/6) then we always have (1 − 4δ)4δ ≥ (1 − 2δ)2δ, and also −4δ ln(4δ) ≥ −2δ ln(2δ), in which case the entropy of the HSR is higher than that of the CSR (equality being attained for δ = 0. Thus, in general:

(A45) $$S_{HSR}\geq S_{CSR}$$

We can therefore see that, in general, the HSR has a higher MaxEnt value than the CSR; which means from a Bayesian perspective that the HSR embodies *fewer* (implicit) assumptions than the CSR. The specific relevant assumption discussed in this paper (and now explicitly evaluated here by this entropic analysis) is that the HSR makes no presumptions about the presence (or absence) of recursion, whereas the CSR a priori excludes recursion as a possibility and thereby introduces this as an additional informational constraint. The CSR therefore has a lower intrinsic entropy than the HSR.

### Appendix A.6. A Sum Rule for Finite and Infinite Impulse Response Filters

Filtering is an important aspect of most modern electronic processing systems, and is generally described using the theory of “digital signal processing” (**DSP**). Such DSP filters are divided into two main classes, which may be considered either as an “infinite impulse response” (**IIR**) or a “finite impulse response” (**FIR**) filter. Photonic device technologies used in digital telecommunications have also borrowed heavily from DSP theory which has been applied to develop important optical components used in optical fibre communications [47]. In particular, Parker et al. [48] state (citing [47]): “*it is known that the ‘Arrayed Waveguide Grating’ (AWG) is a FIR filter, in contrast to the ‘Fiber Bragg Grating’ (FBG) which is an IIR device*”, and they also show that the AWG and FBG filters (both important photonic devices) can be shown to be isomorphic to each other in a particularly simple mathematical relationship.

Considering only the first summation of the CSR of Equation (A34), we can see that the CSR is isomorphic to the general equation for a “finite impulse response” (**FIR**) filter:

(A46) $$Z\left(z\right)=\sum_{n=0}^{N}b_{n}z^{-n}$$

where *Z* is the transfer function: that is, the (complex-valued) frequency response of the filter. The mathematical form of Equation (A46) is equivalent to a discrete Fourier transform (**DFT**) since, as before for Equation (13), the (complex) parameter *z* is given by *z* ≡ exp(i*ω*), in normalised frequency units, and the parameters *b_n_* (which subsume the other summation terms of Equation (A34)) act as Fourier coefficients. The transfer functions of FBG and AWG photonic devices are related through the hyperbolic tangent (*tanh*) function [48]:

(A47) $$Z_{FBG}\left(\omega \right)\equiv \tanh\left[Z_{AWG}\left(\omega \right)\right]$$

What this means is that the IIR response of an FBG can be generated by taking the hyperbolic tangent of the FIR response of an equivalent AWG. In the case of a ‘weak’ IIR filter response (that is, a filter with little feedback) the small argument approximation (tanh *x* ≃ *x* as *x*→0) means that the IIR and FIR become indistinguishable. This is analogous to the “First” (high energy) Born Approximation of quantum mechanics in which the spectral response of a scattering process is closely given by the Fourier transform of the spatial distribution describing a weak potential variation.

It is interesting that the IIR function of Equation (A47) is described using a hyperbolic tangent function in the same way as seen in Appendix A.3, Equation (A11b). This has the implication that there is an IIR filter *sum rule* consisting of the simple concatenation of different FIR filters within an overall hyperbolic tangent function, which is also of DSP engineering interest. For example, consider two different FIR functions *Z_FIR_*_1_ and *Z_FIR_*_2_, each associated with a different IIR function via the following:

(A48) $$Z_{IIRn}=\tanh\left(Z_{FIRn}\right)\;\mathrm{for}\;n=1,2$$

For the concatenation of the two FIR functions it is clear that we can write the following:

(A49) $$Z_{IIR(1+2)}=\tanh\left(Z_{FIR1}+Z_{FIR2}\right)=\frac{\tanh\left(Z_{FIR1}\right)+\tanh\left(Z_{FIR2}\right)}{1+\tanh\left(Z_{FIR1}\right)\tanh\left({HZ}_{FIR2}\right)}$$

(A50) $$Z_{IIR(1+2)}=\frac{Z_{IIR1}+Z_{IIR2}}{1+Z_{IIR1}Z_{IIR2}}$$

Thus, the generic IIR filter (e.g., as similarly seen in Equation (13)) is seen to be also isomorphic to the HSR.

The interpretation and application of Equation (A50) for DSP engineering and filter design remains the work of future research. In the same way that the CSR is *not* a quotient function (in contradistinction to the HSR of Equation (8)) the same applies to the generic mathematical form for a FIR *Z*-transform transfer function given by Equation (A46). And while DSP design of complex yet stable filters will often employ concatenation of multiple FIR filters, as shown in Appendix A.5 so also can the CSR be concatenated to enable multiple hypotheses to be successfully and analytically combined into a single, well-behaved probabilistic sum rule expression.
